# Bioactive Mineralized Cell-Derived Extracellular Matrix via Polymer-Induced Liquid Precursor Enhances Osteogenesis and Bone Regeneration

**DOI:** 10.34133/bmr.0410

**Published:** 2026-09-01

**Authors:** Jae Won Kwon, Jin Jeon, Seung Won Yang, Youngdoo Chung, Jin Hee Park, Yoon Ki Joung, Hee Seok Yang, Kwideok Park

**Affiliations:** ^1^ Center for Biomaterials, Korea Institute of Science and Technology (KIST), Seoul 02792, Republic of Korea.; ^2^ Division of Bio-Medical Science and Technology, KIST School, University of Science and Technology (UST), Seoul 02792, Republic of Korea.; ^3^ Department of Nanobiomedical Science & BK21 FOUR NBM Global Research Center for Regenerative Medicine, Dankook University, Cheonan 31116, Republic of Korea.; ^4^ Department of Biomedical Sciences & Biosystems, Dankook University, Cheonan 32226, Republic of Korea.

## Abstract

Effective bone regeneration requires biomaterials that exhibit appropriate bioactive functions, particularly osteoconductive and osteoinductive properties. Here, we engineered a cell-derived, decellularized extracellular matrix (cdECM) into a novel mineralized ECM scaffold by harnessing the polymer-induced liquid precursor (PILP) process, an effective strategy for generating calcium phosphate (CaP) mineralized constructs. Mineral deposition within cdECM was successfully achieved through the PILP mineralization, which stabilizes the amorphous precursor phase and promotes matrix-associated mineralization. The resulting mineralized ECM (mECM) exhibited osteoconductive properties, as evidenced by excellent cytocompatibility and enhanced cell proliferation of osteogenic cells. The mECM also demonstrated osteoinductive potential, as confirmed by enhanced alkaline phosphatase activity, increased calcification, and up-regulated osteogenic gene expression in mouse preosteoblasts and human mesenchymal stem cells. Moreover, mECM promoted M2-like macrophage polarization and enhanced tubular formation of endothelial cells. To enable localized in vivo delivery of both ECM-derived biological cues and minerals, a sheet-type mECM scaffold was fabricated using hyaluronic acid as a supporting matrix and further stabilized by glutaraldehyde vapor crosslinking. In a mouse calvarial defect model, the mECM sheet facilitated new bone formation and supported advanced bone maturation, accompanied by enhanced angiogenesis and an M2-dominant anti-inflammatory milieu at an early time point. Collectively, our findings demonstrate that PILP mineralization can be successfully applied to cdECM for generating a bioactive mECM scaffold with enhanced regenerative capacity, representing a promising biomaterial platform for bone tissue regeneration.

## Introduction

Bone is a hard tissue that provides structural support, protects organs, and facilitates movements. It is composed of collagen-rich organic matrices reinforced with calcium phosphate (CaP) minerals, enabling structural support and continuous remodeling essential for bone tissue homeostasis [1]. Bone defects arise from a variety of etiologies and often exhibit limited intrinsic healing capacity, owing to complex architecture of bone tissue and the intricacy of its regenerative cascade. As a result, unresolved bone defects result in long-term functional impairment and impose a substantial global health and socioeconomic burden. Bone regeneration typically progresses through well-orchestrated, sequential stages: an initial pro-inflammatory phase with coagulation, anti-inflammatory, and angiogenic phase, followed by soft callus formation, hard callus formation, and finally the remodeling stage [2], in which diverse cell populations dynamically interact and coordinate to regulate and sustain the intrinsic healing process. However, the intrinsic regenerative capacity of bone is constrained by various factors, including defect size, patient age, or lifestyle. Consequently, a wide range of biomaterial-based and tissue engineering strategies has been developed to promote effective bone regeneration [3,4].

Among diverse biomaterials used for bone regeneration, CaP-mineralized materials have attracted considerable attention due to their ability to approximate the composition and hierarchical architecture of native mineralized tissues [5]. Biomineralization refers to the biologically controlled process by which living organisms induce mineral formation, yielding materials with enhanced mechanical strength, durability, and biocompatibility. One bioinspired synthetic approach that emulates this process is the polymer-induced liquid precursor (PILP) method [6]. The PILP process employs negatively charged polymers to sequester ions into a fluidic amorphous calcium phosphate (ACP) precursor, enabling dense and homogeneous intrafibrillar infiltration. Previous studies have demonstrated that the PILP process can be successfully applied to natural scaffolds, such as collagen, dentin, chitosan, and gelatin, as well as synthetic scaffolds, including aliphatic polyesters and polyacrylamide, and even osteoporotic bone to enhance remineralization [7,8]. While traditional biomimetic strategies often aim to generate highly stable crystalline hydroxyapatite (HAp), fully mature phase hyaluronic acid (HA) would degrade very slowly to be synchronized with rapid host tissue remodeling. Alternatively, the metastable ACP phase is often desirable in tissue engineering, because its rapid dissolution releases localized, osteoinductive ions that would accelerate early bone regeneration [9].

Meanwhile, extracellular matrix (ECM) is a complex and highly organized network of macromolecules composed primarily of fibrous proteins, such as collagen, fibronectin, and laminin, together with proteoglycans and extracellular vesicles. The ECM provides essential mechanical support and biochemical cues to resident cells, and is typically obtained from animal tissues through decellularization processes [10]. Due to its ability to recapitulate the native microenvironment, ECM has emerged as an attractive biomaterial in tissue engineering, offering a cell-free platform that promotes tissue regeneration [11]. Over recent years, cell-derived ECM (cdECM) has also gained increasing attention, proving its versatility and regenerative potential. cdECM is produced from in vitro cultured cells, enabling more simplified and reproducible decellularization and sterilization process than tissue-derived ECM (tdECM) [12]. The cdECM is typically obtained as a tunable 2-dimensional (2D) matrix, allowing facile integration with other biomaterials. In addition, the cdECM itself has demonstrated considerable regenerative potential across a wide range of biomedical applications [13,14]. Among diverse cell populations, mesenchymal stem cells (MSCs), especially umbilical cord-derived mesenchymal stem cells (UCMSCs), have been employed in previous studies because of their superior expansion capacity and robust ECM production [15]. ECM-based biomaterials have been widely employed in bone regeneration, particularly due to intrinsic bioactivity and biomimetic compositions. In addition, noncollagenous proteins (NCPs) in the ECM play key regulatory roles in bone mineralization with their relatively low quantities [16,17]. Given its controllability and accessibility, cdECM represents a promising source that can be prepared from autologous or allogenic cells under standardized culture conditions. Based on these attributes, we hypothesized that cdECM would serve as an effective substrate for PILP mineralization, and that the resulting mineralized ECM (mECM) could exhibit enhanced osteogenic potential.

In this study, mECM was engineered as a bioactive scaffold for bone regeneration using a PILP mineralization approach. Negatively charged polymers were employed to stabilize and arrest the mineral phase in its highly reactive amorphous state within the cdECM. The cdECM was obtained from cultured UCMSCs and subsequently mineralized via the PILP process. The osteoconductive and osteoinductive potential of the resulting mECM was validated through in vitro cytocompatibility assays and osteogenic differentiation studies using mouse preosteoblasts and human MSCs (hMSCs). For in vivo application, the mECM was further processed into a sheet-type scaffold by incorporating HA, followed by additional crosslinking. The therapeutic efficacy of the mECM sheet was then assessed in a murine calvarial defect model by evaluating the various indicators of bone regeneration. Collectively, this study proposes a new application of PILP mineralization toward cdECM and highlights mECM as a promising composite biomaterial for bone regeneration.

## Materials and Methods

### Mineralization of cdECM using PILP process

#### Cell culture and decellularization

Human UCMSCs (UCMSC P8; CEFO UCMSC, CEFObio, Seoul, Republic of Korea) were cultivated on tissue culture plate (TCP) at a density of 1.5 × 10^4^ cells/cm^2^ in CEFOgro Human MSC growth medium (CEFOgro-MSC, CEFObio) containing 50 ml of fetal bovine serum (FBS) and 2.5 ml of penicillin and streptomycin (p/s). The medium was changed every 2 to 3 d. On days 6 and 7, those confluent cells were washed with phosphate-buffered saline (PBS) and subjected to decellularization by dispending a solution of 0.25% Triton-X 100 and 20 mM NH_4_OH into the plates and subsequent treatment with 50 U/ml deoxyribonuclease (DNase) I (18047-019; Invitrogen, MA, USA) and 2.5 μl/ml ribonuclease (RNase) A (12091-039; Invitrogen) for 1 h at 37 °C. After several washing with PBS, resulting cell-derived, decellularized extracellular matrix (cdECM) was kept at −20 °C in deionized water (DW) for further use. The entire process was performed in a sterile condition.

#### Optimization of PILP mineralization conditions for cdECM

For the PILP process, poly aspartic acid (pAsp) was selected as the process-directing agent. Poly-(α, β)-dl-aspartic acid sodium salt (P3418, Sigma-Aldrich, US) and calcium chloride dihydrate (CaCl_2_·2H_2_O) were dissolved in 100 mM tris buffer (TB; pH 7.4, 648315, Merck, Germany). Separately, di-potassium hydrogen phosphate trihydrate (K_2_HPO_4_·3H_2_O, P5504, Sigma-Aldrich) was dissolved in 100 mM TB. To optimize the PILP process on cdECM, mineralization solution was prepared at different concentration as follows: 1× (25 μg/ml pAsp, 5 mM CaCl_2_·2H_2_O, and 2.5 mM K_2_HPO_4_·3H_2_O), 5× (125 μg/ml pAsp, 25 mM CaCl_2_·2H_2_O, and 12.5 mM K_2_HPO_4_·3H_2_O), 10× (250 μg/ml pAsp, 50 mM CaCl_2_·2H_2_O, and 25 mM K_2_HPO_4_·3H_2_O), and 20× (500 μg/ml pAsp, 100 mM CaCl_2_·2H_2_O, and 50 mM K_2_HPO_4_·3H_2_O). Equal volume (1:1) of each mineralization solution was used and mixed in cdECM plates. The total volume was adjusted according to the culture vessel size: 5 ml for 100-mm dish, 1 ml for 6-well plate, and 500 μl for 12-well plate. Each mineralization solution was applied to cdECM samples and incubated at 37 °C for 1, 2, or 3 weeks, followed by several washes with DW. The mECM samples then were observed under a light microscope, and the deposited minerals were subsequently decalcified for further analyses.

For mineral quantification, decalcification was performed using 0.5 M hydrochloric acid (HCl; H9892, Sigma-Aldrich) at room temperature (RT) overnight under gentle shaking. The decalcified solutions were collected and centrifuged at 25,000*g* for 5 min at 4 °C. The resulting supernatants were transferred to fresh tubes and stored at 4 °C until analysis. Prior to the assays, the samples were diluted 1:100 in 1× tris-buffered saline (TBS) (pH 7.4) to match the detection range of assays and neutralize the pH. The diluted samples were freshly prepared and used within 30 min to prevent reprecipitation. Calcium and phosphate were quantified using a colorimetric calcium assay kit (ab102505, Abcam) and phosphate assay kit (ab65622, Abcam), respectively, according to the manufacturer’s instructions. Quantitative results were calculated based on the standard curve and presented as mineral weight (μg).

#### Preparation of PILP-induced mECM

An optimized condition of PILP mineralization was determined to be 10× concentration applied to cdECM for 1 week. Briefly, cdECM was prepared by decellularization, during which it remained adherent to 100-mm culture dishes. Two mineralization solutions were separately prepared in 1× TBS: one containing 250 μg/ml poly aspartic acid (pAsp) and 50 mM CaCl_2_·2H_2_O, and other containing 25 mM K_2_HPO_4_·3H_2_O. The pAsp/CaCl_2_ solution (2.5 ml) was first applied to cdECM dishes. After removing air bubbles, the K_2_HPO_4_ solution (2.5 ml) was slowly added and gently mixed. The treated cdECM was subsequently incubated at 37 °C under static conditions for 7 d to induce mineralization. During incubation, the pH of the mineralization solution was monitored using pH indicator paper (1.09557, pH range 6.4 to 8.0, MQuant, Germany). Following incubation, the resulting mECM was washed at least 5 times with DW. To confirm the presence of residual ions, the mECM was further incubated at 37 °C in DW for 24 h, and the residual ions were quantified using the collected supernatant. Both ECM (nonmineralized) and mECM were separately collected in 1× TBS (pH 7.4, TR2005-100-74, Biosesang, Korea) using a cell scraper and homogenized with an ultrasonicator to obtain uniform suspensions. The ECM and mECM suspensions were further solubilized prior to protein quantification using a bicinchoninic acid (BCA) protein assay (23225, Thermo Fisher Scientific). Specifically, to enhance protein solubility, a urea buffer consisting of 7 M urea supplemented with protease inhibitors in 1× radioimmunoprecipitation assay (RIPA) buffer was prepared, and the ECM and mECM suspensions were diluted at 1:10 in the buffer. The samples were subsequently sonicated to maximize solubilization and centrifuged at 16,000*g* for 10 min at 4 °C. The resulting supernatants were collected and further diluted 1:10 in 1× TBS prior to analysis. Based on the quantified proteins, the ECM and mECM suspensions were adjusted to 1% (w/v; 10 mg/ml) in 1× TBS and stored at 4 °C for short-term or freeze-dried for long-term purpose. These suspensions were subsequently used for in vitro studies or for fabrication of sheets for in vivo transplantation.

### Characterization of mECM

#### Fourier transform infrared spectroscopy

The molecular compositions of ECM and mECM were separately evaluated using Fourier transform infrared (FT-IR) spectrophotometer (Nicolet 560, Nicolet Co., Madison, WI, USA). The samples were freeze-dried at −80 °C. All the FT-IR spectra were recorded in the wavelength ranges of 500 to 4,000 cm^−1^, with the resolution of 4.0 cm^−1^ and 32-times scanning.

#### X-ray photoelectron spectroscopy

The chemical composition of mECM was assessed using x-ray photoelectron spectroscopy (XPS; NEXSA, Thermo Fisher Scientific, MA, USA). Both ECM and mECM samples were prepared on plastic film and completely dried by freeze-drying at −80 °C overnight. XPS measurements were conducted using a monochromated Al Kα x-ray source (photon energy = 1,486.6 eV, power = 72 W) at a take-off angle of 0°. The binding energy scale was calibrated with reference to the C–C peak at 284.8 eV as a reference.

#### Scanning electron microscopy and energy-dispersive spectroscopy

The morphology and elemental distribution of the mECM were analyzed by field-emission scanning electron microscopy (FE-SEM) and energy-dispersive spectroscopy (EDS). Samples were completely dried by freeze-drying at −80 °C overnight. FE-SEM images were obtained using a Teneo Volumescope (FEI, Lausanne, Switzerland) operated at 10 kV. Subsequently, EDS analysis was performed by EDAX Octan Elect Super (AMETEK, Pennsylvania, USA) equipped on the FE-SEM. Elemental distribution was analyzed using EDAX APEXTM EDS software.

#### Human angiogenesis array of ECM and mECM

Proteome Profiler human angiogenesis array (ARY007; R&D Systems, MN, USA) was used to profile angiogenesis-related factors in the ECM and mECM. Briefly, the nitrocellulose membrane containing 55 angiogenesis-related antibody spots was blocked with the supplied blocking buffer and then incubated overnight at 4 °C with ECM and mECM suspension (1 mg/ml), respectively, along with a biotinylated detection antibody cocktail. After being washed with the provided wash buffer, streptavidin–horseradish peroxidase and chemiluminescent detection reagents were sequentially applied to the membrane. Chemiluminescence signals were captured using an iBright CL1500 imaging system. The densitometric quantification was performed using iBright analysis software, and the results are presented as the relative ratio (%) of the positive dots normalized to the reference dots (positive control).

### Biocompatibility and cellular activation of mECM

#### In vitro dose dependence of mECM

To optimize the mECM concentration for in vitro studies, mECM solutions were prepared at various concentrations of 1, 5, 10, 50, and 100 μg/ml in medium. Mouse preosteoblasts [MC3T3-E1; American Type Culture Collection (ATCC), VA, USA] were seeded at a density of 1 × 10^4^ cells/cm^2^ in minimum essential medium α (MEM-α, A10490-01; Gibco) supplemented with 10% FBS, 100 U/ml penicillin, and 100 μg/ml streptomycin (p/s). After overnight incubation, the medium was replaced with fresh one containing the given concentrations of mECM, while cells cultured in normal medium without mECM served as a control group. After 3 d, cytotoxicity was evaluated using a Cell Counting Kit-8 (CCK-8) assay (CK04; Dojindo, Kumamoto, Japan), and the optical density (OD) was measured at 450 nm.

In parallel, the osteogenic effects of mECM at different concentrations were also evaluated using MC3T3-E1 cells. The cells were seeded at a density of 5 × 10^4^ cells/cm^2^ in MEM-α supplemented with 10% FBS and p/s under standard culture conditions (37 °C, 5% CO_2_). After overnight incubation, the cells were washed with PBS and cultured in osteogenic differentiation medium (ODM; MEM-α containing 100 μg/ml ascorbic acid and 5 mM β- glycerophosphate) containing the given concentrations of mECM. The medium was refreshed every 2 to 3 d for 11 d, and cells were fixed with 4% paraformaldehyde on day 11. The fixed cells were washed several times with DW and stained with 40 mM alizarin red S (ARS) solution. For quantitative analysis, the bound ARS was eluted using 10% acetic acid, and the OD value was measured at 405 nm.

#### Cell viability and proliferation assay

To evaluate cell viability, hMSCs (PT-2501; Lonza, P6) were seeded on TCP at a density of 2 × 10^4^ cells/cm^2^ in Dulbecco’s modified Eagle’s medium (DMEM; 11995-065; Gibco, MT, USA) supplemented with 10% FBS and p/s under standard culture conditions (37 °C, 5% CO_2_). Mouse preosteoblasts were also cultivated at a density of 5 × 10^3^ cells/cm^2^ MEM-α using identical supplements and conditions. After overnight incubation, the medium was replaced with fresh medium containing 10 μg/ml of ECM and mECM, respectively. After 2 d of culture, cell viability was assessed using LIVE/DEAD Viability/Cytotoxicity Kit (L&D, L3224; Invitrogen). Briefly, the live cells were stained with 2 μM calcein-AM (green) and dead cells were stained with 4 μM ethidium homodimer-1 (EthD-1; red) for 30 min. The stained cells were observed using a confocal laser scanning microscope (Carl Zeiss). For cell proliferation analysis, cells were cultivated overnight under the same conditions, and then the medium was changed with a fresh medium containing 10 μg/ml of ECM and mECM, respectively. Cell proliferation was quantified at 1, 4, and 7 d (*n* = 3 per group) using the CCK-8 assay. The results were expressed as OD value measured at 450 nm.

#### Observation of macrophage polarization

To assess macrophage polarization, mouse macrophage cells (RAW 264.7; ATCC) were cultured on TCP at a density of 2 × 10^4^ cells/cm^2^ in DMEM supplemented with 10% FBS and p/s under standard culture conditions (37 °C, 5% CO_2_). After overnight incubation, the medium was replaced with fresh medium containing 10 μg/ml of ECM or mECM, while 100 ng/ml of lipopolysaccharide (LPS; L2630, Sigma-Aldrich, MA, USA) was used as a control. Cell lysates were harvested after 24 h of incubation and analyzed for polarization by quantitative reverse transcription polymerase chain reaction (RT-qPCR). Meanwhile, to obtain conditioned medium (CM), the treated cells were washed with PBS and incubated in serum-free medium (SFM) for an additional 24 h. The resulting CM was collected and used for tubular formation assay. In addition, macrophages were costimulated using LPS and ECMs (ECM or mECM) to evaluate their modulatory effects on LPS-induced inflammatory responses. After cell seeding and subsequent incubation (24 h), the medium was replaced with fresh one containing 100 ng/ml of LPS and 10 μg/ml of ECMs. After 24 h of cotreatment, total RNA was extracted and analyzed by RT-qPCR.

#### HUVEC tubular formation assay

The pro-angiogenic potential of the CM derived from the macrophages cultivated with ECM or mECM was examined via a tube formation assay using human umbilical vein endothelial cells (HUVECs; CRL-1730, ATCC, P4). Briefly, 96-well plates were coated with 50 μl of Matrigel and allowed to polymerize at 37 °C for 30 min. HUVECs were then seeded onto the Matrigel layer at a density of 2 × 10^4^ cells/well, after being resuspended in the CM from each experimental group. The plates were incubated for 15 h at 37 °C in a 5% CO₂ atmosphere to facilitate the development of capillary-like networks. After calcein-AM staining for 30 min, the tubular structures were visualized using a fluorescence microscope. For quantitative analysis, the captured images were analyzed with ImageJ software to measure key angiogenic parameters, including the number of meshes, number of junctions, total mesh area, and the number of segments.

### Osteogenic differentiation potential of mECM

#### Induction of osteogenic differentiation

The osteogenic activity of mECM was assessed using MC3T3-E1 and hMSCs, respectively, for osteogenic differentiation in vitro. MC3T3-E1 cells were seeded at a density of 5 × 10^4^ cells/cm^2^ in MEM-α supplemented with 10% FBS and p/s under standard culture conditions (37 °C, 5% CO_2_). After overnight incubation, the cells were washed with PBS, and the ODM (MEM-α containing 100 μg/ml of ascorbic acid and 5 mM β- glycerophosphate) was applied and refreshed every 2 to 3 d for 21 d. The hMSCs were seeded at a density of 2 × 10^4^ cells/cm^2^ in low-glucose DMEM (11885-084; Gibco) supplemented with 10% FBS and p/s under the same culture condition. When the cells reached approximately 80% confluence, they were washed with PBS, added with ODM (A1007201; StemPro, Gibco), and maintained for 14 d, with the medium change every 2 to 3 d. In the experimental groups, 10 μg/ml of ECM or mECM in suspension was separately added to the ODM.

#### ARS staining and quantification

Osteogenic induction of each cell was evaluated using an ARS staining quantification assay (8678; ScienCell Research Lab, CA). Briefly, the cells were fixed with 4% paraformaldehyde for 15 min. After being washed with DW, they were incubated in 40 mM ARS staining solution for 30 min at RT. Following several washes with DW, high-resolution images were acquired using an inverted microscope (Axio Vert.A1; Carl Zeiss, Oberkochen, Germany). For quantification of ARS, the stained cells were incubated in 10% acetic acid 30 min at RT and transferred to tubes. The samples were homogenized to form a slurry and centrifuged at 20,000*g* for 15 min. The supernatants were neutralized with 10% ammonium hydroxide, and the absorbance was measured at 405 nm using a microplate reader. The ARS standard was prepared according to the manufacturer’s instructions, and the results were calculated based on the ARS concentration (mM) determined from standard curve.

#### Alkaline phosphatase staining and enzyme activity assay

Alkaline phosphatase (ALP) activity of each cell type was evaluated using both staining and enzyme activity assay. The cells were washed with PBS and fixed with 4% paraformaldehyde for 5 min at RT. After washing, the fixed cells were then incubated with 5-bromo-4-chloro-3-indolylphosphate/nitro blue tetrazolium liquid substrate (BCIP/NBT; B1911; Sigma-Aldrich) for 1 h at RT in the dark. The stained cells were washed with DW and observed under a microscope. ALP activity was further quantified using an Alkaline Phosphatase Assay Kit (ab83369; Abcam, Cambridge, UK). Briefly, the cells were homogenized in ALP assay buffer I provided in the kit and centrifuged at 15,000 rpm for 15 min. The supernatants were incubated with p-nitrophenyl phosphate (pNPP) substrate at RT for 1 h in the dark, and the reaction was terminated with the stop solution. After gentle shaking, the OD value was measured at 405 nm on a microplate reader. The standard curve was prepared according to the manufacturer’s instructions. ALP activity was calculated based on standard curve and expressed as mU/ml.

### Quantitative reverse transcription polymerase chain reaction

For gene expression analysis, total mRNA was extracted from the MC3T3-E1 and RAW 264.7 treated with ECM or mECM at each time point using QIAzol Lysis reagent (Qiagen, Hilden, Germany), according to the manufacturer’s instruction. The concentration and purity of the extracted RNA were determined at 260 nm using a NanoDrop spectrophotometer (ND-1000; Thermo Fisher Scientific). The cDNA was synthesized from the extracted RNA using a SuperScript VILO cDNA Synthesis Kit (11754050; Thermo Fisher Scientific). RT-qPCR was performed using TB Green Premix Ex Taq (RR420A, TaKaRa, Japan) and the primers specific to the target genes on a QuantStudio 5 Real-Time PCR instrument (Applied Biosystems, MA). Gene expression levels were normalized to Gapdh expression and calculated using the 2^−ΔΔCt^ method. Results were expressed as fold changes relative to the control group (set to 1). All experiments were performed in triplicate. Primer sequences are provided in Table 1.

**Table 1.** Primer sets used in RT-qPCR

Gene	Forward sequence (5′→3′)	Reverse sequence (5′→3′)
*Gapdh*	TGACAACTTTGGCATTGTGGAA	GGATGCAGGGATGATGTTCTG
*Cd80*	ATACGACTCGCAACCACACC	GAATCCTGCCCCAAAGAGCA
*Nos2*	GTGACCATGGAGCATCCCAA	GGTGCCCATGTACCAACCAT
*Arg1*	CTTGCGAGACGTAGACCCTG	TCCATCACCTTGCCAATCCC
*Cd206*	GTGGGGACCTGGCAAGTATC	CACTGGGGTTCCATCACTCC
*Bglap*	CCCAGACCTAGCAGACACCA	GCCGGAGTCTGTTCACTACC
*Spp1*	CCAACGGCCGAGGTGATAG	GGACTCCTTAGACTCACCGC
*Runx2*	GGCCACTTACCACAGAGCTA	GCCCTAAATCACTGAGGCGA
*Alpl*	GGGCAATGAGGTCACATCCA	GTGGTTCACCCGAGTGGTAG
*Sp7*	ACAGCCAACCCTAGCCTACC	GTTTTGGGGGCTGAAAGGTC

### Fabrication of mECM sheet and characterization

#### Fabrication of mECM sheet

For sheet fabrication, sodium hyaluronate (HA 2.0; HYUNDAI BIOLAND, Cheongju, Republic of Korea) was homogenized in either ECM or mECM suspension at a final concentration of 3% (w/v) using a homogenizer and allowed to dissolve overnight at 4 °C. The resulting ECM or mECM slurry was poured into molds and frozen at −80 °C overnight. The frozen samples were then freeze-dried at −80 °C under 5-ppm (parts per million) pressure for 24 h. The resulting sheets were stored in a vacuum desiccator until further use. To enhance the mechanical property of the sheets, they were trimmed to the desired size using a biopsy punch or scissors and then subjected to crosslinking by the exposure to 25% glutaraldehyde (GA; G6257; Sigma-Aldrich) vapor for 15 h under vacuum at RT. After then, the residual GA was removed by vacuum drying for 3 d at RT. The resulting GA-crosslinked sheets were subsequently stored in a vacuum desiccator. The amount of residual GA was quantitatively analyzed using the MBTH (3-methyl-2-benzothiazolinone hydrazone) assay. Each vacuum-dried scaffold was immersed in 1 ml of phosphate-buffered saline (PBS) and incubated at 37 °C for 24 h. The supernatants were collected and filtered through a 3-kDa molecular weight cutoff centrifugal filter (Amicon Ultra-4, Merck Millipore). The filtrate was then reacted with MBTH reagent (Sigma-Aldrich), and the absorbance was measured at 620 nm. The concentration of residual GA was calculated and normalized against non-crosslinked control samples.

#### Micro-computed tomography analysis

Minerals of mECM sheet were detected using micro-computed tomography (CT). Both ECM and mECM sheet were scanned using a Skyscan 1176 system (Bruker, Kontich, Belgium). Scan parameters were set to a source voltage of 36 kV and a current of 222 μA, without filter. Following acquisition, the projection data were reconstructed into 3D images using NRecon software.

#### Rheological properties

A rheometer (MCR102; Anton Paar, Austria) equipped with a 25-mm-diameter parallel plate and a sample gap of 0.5 mm was used for viscosity assessment. All the samples were prepared as liquid foams based on HA concentrations (1%, 2%, and 3%) with or without mECM. Viscosity was measured by increasing the shear rate at RT (25 °C) over a range of 0.1 to 100 rad/s. For shear stress sweep test, GA-crosslinked and non-crosslinked sheets (10 mm diameter) were prepared and allowed to absorb DW for 30 min at RT prior to analysis. Shear stress sweeps were performed with increasing oscillatory frequency. The storage (*G*′) and loss (*G*″) modulus were measured at 1% shear strain at RT using a frequency sweep test over a range of 1 to 100 rad/s. All the rheological data were processed using RheoCompass software.

#### Characterization of ECM and mECM sheet

According to the degree of GA crosslinking, the molecular compositions were determined using FT-IR (Nicolet 560; Nicolet Co). The sheets were prepared in 3 groups: non-crosslinked, GA-crosslinked 0.5% ECM, and GA-crosslinked 1% ECM, each with 2 mm diameter. FT-IR spectra were obtained in the wavelength ranges of 500 to 4,000 cm^−1^, with a resolution of 4.0 cm^−1^ and 32 scans. The stability of the sheets was also evaluated by a swelling test using GA-crosslinked and non-crosslinked sheets (5 mm diameter). Each sheet was covered with 100 μl of DW, and additional DW was added every 2 min for a total of 30 min at RT. The hydrated samples were visually examined for morphological changes and tested for their stability using forceps. In addition, the cytotoxicity of GA-crosslinked sheet was evaluated by cell viability and CCK-8 assays. Once MC3T3-E1 cells were seeded at 1 × 10^4^ cells/cm^2^ in MEM-α with the same supplements and then incubated overnight, GA-crosslinked and non-crosslinked sheets (2 mm diameter) were placed in transwell inserts with 8.0-μm pore size (FALCON) and loaded onto the culture plate. The culture medium was replaced with fresh medium. After 3 d of culture, cell viability and CCK-8 assays were performed as described in the In vitro dose-dependence of mECM section. The nontreated group (NT) served as a positive control.

### Transplantation of mECM sheet and analysis

#### Transplantation of mECM sheet into calvarial defect model

Eight-week-old female ICR mice, obtained from Dayun (Gyeonggi-do, Korea), were used for this study. The animal study was approved by the Institutional Animal Care and Use Committee at Dankook University (DKU-23-052). After anesthesia via an intraperitoneal injection of a ketamine and xylazine mixture (4:1, v/v), the surgical site was sterilized, and a longitudinal midline incision was made on the cranium to expose the sagittal suture line. Two types of defects were created depending on the designated evaluation time point (*n* = 5 per group). For the mice sacrificed at 1 week, the periosteum was selectively stripped using a surgical trephine burr (XTP3404, Dentium, Seoul, Korea) with continuous sterile saline irrigation to evaluate the initial host response, including early inflammatory reaction and angiogenesis. The samples (HA, ECM, and mECM sheets) were implanted into prepared sites (one sheet per one mouse). In contrast, for the mice sacrificed at 8 weeks, a circular, full-thickness calvarial defect (4 mm in diameter) was created using a surgical trephine burr to assess bone regeneration. The prepared ECM and mECM sheets were implanted into the defect sites (one sheet per one defect). The mice were euthanized via CO_2_ asphyxiation at their respective time points. At 1 week, the samples were retrieved en bloc with the overlying scalp tissue, whereas, at 8 weeks, the calvariae containing the defect sites were harvested. All samples were fixed overnight in 4% paraformaldehyde (PFA; Sigma-Aldrich).

#### Live micro-CT analysis

To longitudinally monitor new bone formation, in vivo micro-CT was performed at 4 and 8 weeks post-implantation. Mice were anesthetized with an intraperitoneal injection of ketamine and xylazine, consistent with the surgical procedure, and then scanned using a Skyscan 1176 system (Bruker, Kontich, Belgium). Scan parameters were set to a source voltage of 50 kV and a current of 500 μA, with a 0.2-mm aluminum filter. Following acquisition, the projection data were reconstructed into 3D images using NRecon software. For quantitative analysis, a cylindrical region of interest (ROI) with 4 mm diameter, corresponding to the original defect area, was defined. Subsequently, the bone volume fraction (BV/TV) and bone mineral density (BMD) within the ROI were calculated using CTAn and CTvol software.

#### Histological analysis

For histological analysis, the harvested calvarial tissues were fixed in 4% PFA at 8 weeks. Following a rinse with PBS to remove residual fixative, the samples were decalcified in Decalcifying Solution-Lite (Sigma-Aldrich) for 2 h at RT. The specimens were dehydrated through a graded ethanol series, then embedded in paraffin, and sectioned into 6-μm-thick slices using a microtome (RM2255, Leica, Bensheim, Germany). Prior to staining, the sections were deparaffinized in xylene, rehydrated through a descending ethanol gradient, and stained with hematoxylin and eosin (H&E) for overall morphological assessment. The stained sections were imaged using an optical microscope to visualize bone regeneration, and the area of newly formed bone and bone density were quantified using ImageJ software (National Institutes of Health, Bethesda, MD). The new bone formation was quantified as the percentage of newly formed bone relative to the original bone defect area. Bone density was also calculated as the percentage of newly formed bone area relative to the total tissue area within the defect, defined as the sum of the new bone area and fibrous tissue area.

#### Immunohistochemical analysis

To detect the expression of osteocalcin (OCN), immunofluorescence staining was performed on the paraffin-embedded sections (*n* = 4 per group). The sections were first deparaffinized in xylene and rehydrated through a graded ethanol series. The sections were permeabilized and blocked with a solution of 5% goat serum and 0.1% Triton X-100 in PBS to prevent nonspecific binding. The samples were then incubated overnight at 4 °C with a primary antibody specific for OCN. Following primary antibody incubation, the slides were washed, incubated with a fluorescein isothiocyanate Rhodamine-conjugated secondary antibody (Jackson Immuno Research Laboratories), and counterstained with 4′,6-diamidino-2-phenylindole (DAPI) to visualize the nuclei. Finally, the stained sections were mounted and imaged using a fluorescence microscope (U-RFLT50, Olympus, Shinjuku, Japan). Quantification of fluorescence intensity was measured using ImageJ software. The signal intensity of the target marker was expressed as a percentage, normalized to that of the DAPI fluorescence intensity.

### Statistical analysis

All the data are presented as mean ± standard deviation, along with individual data points. Statistical analysis was performed using a 2-tailed (α = 0.05) Student’s *t* test for the 2 experimental groups. One-way analysis of variance (ANOVA) with a post hoc Tukey’s multiple comparison test was also carried out for more than 3 test groups. Two-way ANOVA with a post hoc Tukey’s multiple comparison test was performed for more than 3 test groups with 2 or more variables. Statistically marked differences are denoted by *P < 0.05, **P < 0.01, ***P < 0.001, or ****P < 0.0001.

## Results

### Characterization of mECM

**Fig. 1.**
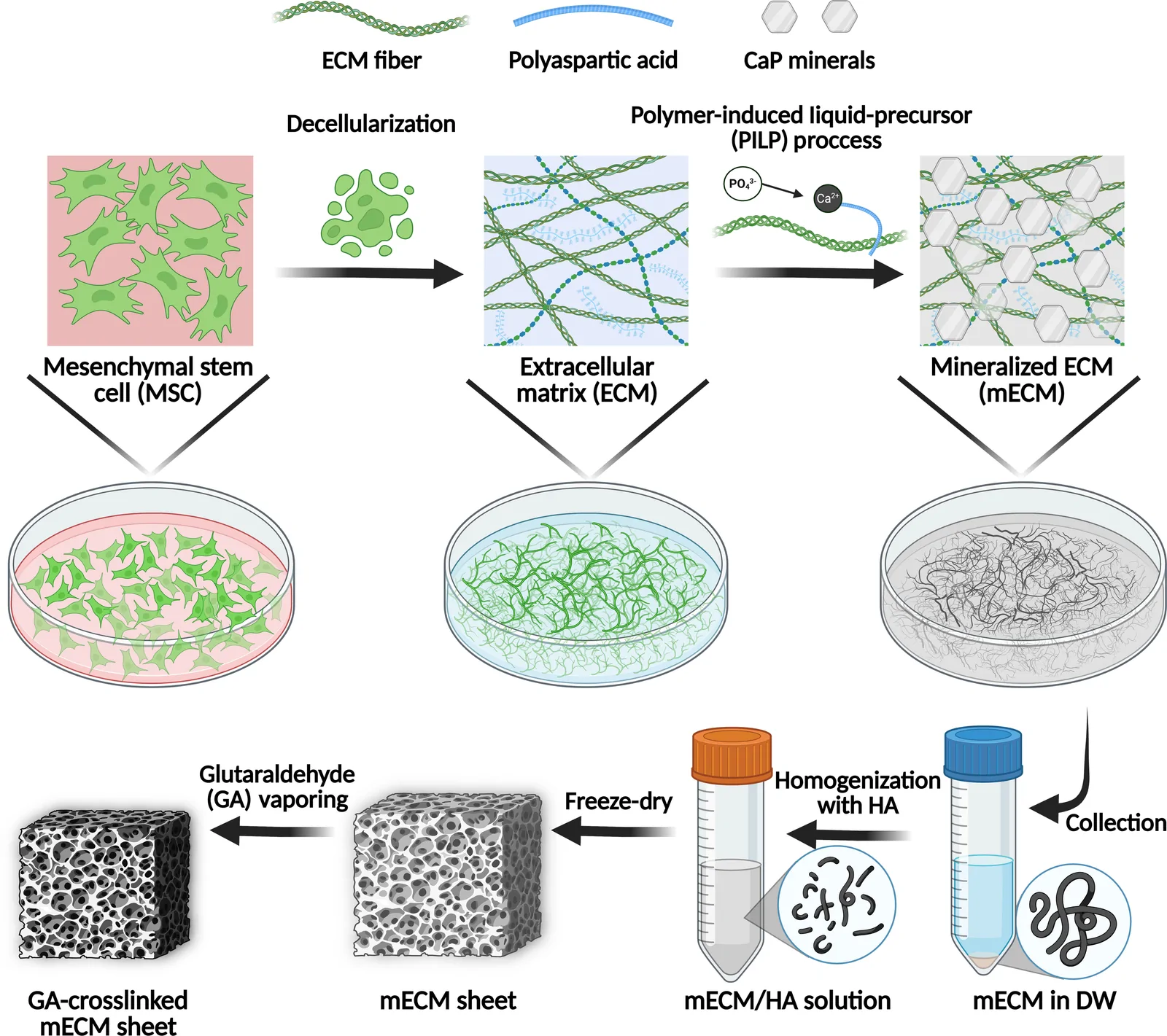
Process of ECM mineralization via PILP and mECM sheet fabrication. cdECM was obtained through decellularization of in vitro cultured MSCs. Mineralization was then induced via PILP process with pAsp and ions. The resulting mECM was collected, homogeneously mixed with HA, freeze-dried, and then finally subjected to the crosslinking using GA to fabricate mECM sheet. Created with Biorender.

The mECM was produced via the PILP process (Fig. 1). Mineralization of the cdECM was optimized by varying the concentrations of the mineralization agents (1× 5×, 10×, and 20×) and the incubation periods (1, 2, and 3 weeks). Microscopic imaging was used to evaluate the homogeneity of CaP mineral deposition (Fig. S1A). After 1 week, ECM fibers were still clearly visible and only sparse mineral deposition was observed under the 1× concentration, whereas the others (5×, 10×, and 20×) exhibited extensive mineral coverage. Notably, the 10× concentration showed homogeneous and complete mineral coverage on the cdECM surface. However, prolonged incubation resulted in mineral detachment and self-aggregation in several regions. In particular, exposure of ECM fibers was observed in the 5×, 10×, and 20× group after 3 weeks, due to mineral detachment or crystallization. The amount of deposited CaP minerals was quantified using calcium and phosphate assay after decalcification (Fig. S1B and C). Calcium deposition was markedly increased in the 5×, 10×, and 20× group compared with the 1× group, but decreased after 3 weeks of incubation. The highest calcium contents were confirmed in the 10×-1wk (310.3 ± 2.8 μg) and 5×-2wk (306.4 ± 2.7 μg) groups (Fig. S1B). Similarly, phosphate deposition increased under higher concentrations and decreased after 3 weeks. The highest phosphate contents were found in the 10×-1wk (535.4 ± 7 μg), 10×-2wk (519.6 ± 4.7 μg), and 5×-2wk (483.7 ± 1.9 μg) groups (Fig. S1C). In addition, ECM detachment was clearly observed after 3 weeks of incubation (Fig. S1D). The total protein content of the resulting mECM was also quantified (Fig. S1E). ECM protein content decreased following the mineralization process, with the 1× condition showing a particularly pronounced reduction to below 40% of native ECM level. In contrast, the 10×-1wk group exhibited the highest ECM preservation (95.14 ± 0.35%). Based on these findings, the 10×-1wk group was selected as the optimal mineralization condition, and mECM generated under this condition was used for all subsequent analyses.

**Fig. 2.**
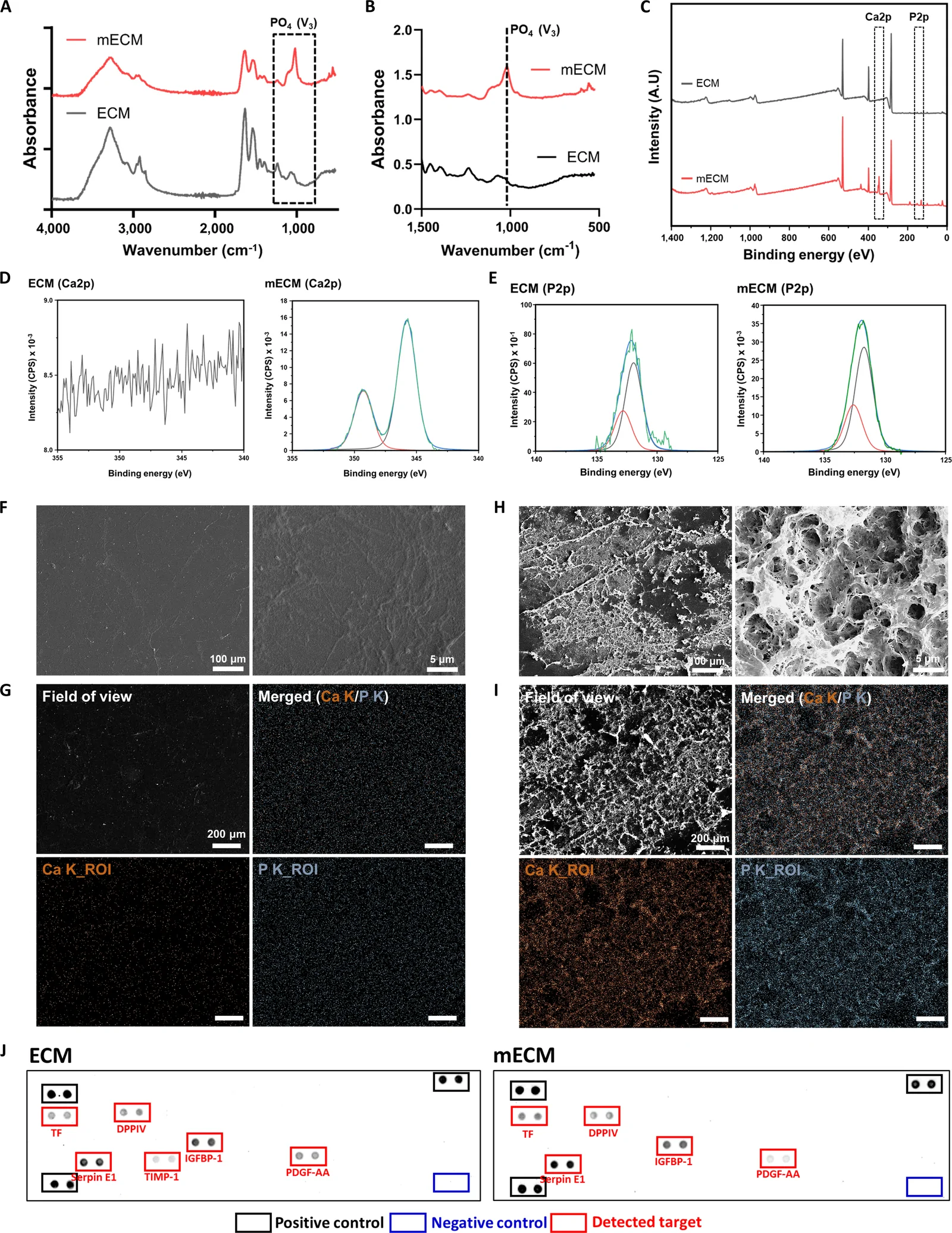
Characterization of mECM. (A) FT-IR spectra of ECM and mECM samples. The boxed region indicates the characteristic peaks corresponding to phosphate groups. (B) Enlarged FT-IR spectra ranging from 1,500 to 500 cm^−1^ highlight the PO_4_ (V_3_) peak around 1,020 cm^−1^ (dotted line). (C) XPS spectra of ECM and mECM show the Ca2p and P2p peaks (boxed regions). Fitted high-resolution XPS spectra of (D) Ca2p and (E) P2p from ECM and mECM, respectively. (F) SEM surface view of ECM. Scale bars, 100 and 5 μm. (G) EDS analysis of ECM displays calcium and phosphorus distribution with field view and merged image. Scale bar, 200 μm. (H) SEM surface morphology of mECM. Scale bars, 100 and 5 μm. (I) EDS elemental mapping of mECM. (J) Profile of human angiogenic factors in the ECM and mECM, respectively.

The mECM was then subject to the analysis of FT-IR, XPS, and SEM–EDS to confirm mineral compositions and deposition. The FT-IR spectra of mECM and ECM showed similar overall patterns, except for a distinct peak appearing around 1,000 cm^−1^ in the mECM spectrum (Fig. 2A). When analyzed narrowly in the range of 500 to 1,500 cm^−1^, a sharp peak at 1,035 cm^−1^ was obvious in the mECM only, corresponding to PO_4_^3−^ vibrations (Fig. 2B) [18]. For the comprehensive analysis of the mECM surface features, XPS and SEM–EDS were carried out. XPS revealed some elemental changes in the mECM that proved an increased level of calcium (Ca) and phosphorus (P), due to mineralization compared with the ECM (Fig. 2C). The narrow-scan XPS spectra showed the appearance of Ca2p peaks exclusively in the mECM (Fig. 2D). Consistent with the FT-IR results, the XPS analysis demonstrated an increased intensity of P2p peaks in the mECM compared with ECM (Fig. 2E). The XPS survey spectra revealed the atomic compositions of ECM and mECM, respectively, with the mECM exhibiting a Ca/P ratio of 1.344 (Table 2). The SEM images of the ECM revealed only fibrous structures (Fig. 2F), whereas the mineral deposits formed along the fibers were observed in mECM (Fig. 2H). High-magnification SEM images showed the progressive mineral deposition on the ECM fibers, which were clearly visible at day 0 but became gradually covered over the treatment period (Fig. S2A). Additionally, EDS elemental mapping revealed only a small amount of P on the ECM surface (Fig. 2G). In contrast, abundant Ca and P deposits were observed on the mECM surface, confirming successful mineralization (Fig. 2I). Consistently, EDS elemental analysis demonstrated the elemental compositions of ECM and mECM, with the mECM retaining the Ca/P ratio of 1.4 (Table 3). For further characterization, the relative proportions of minerals, proteins, and other components in mECM mass disclosed that the ratio of minerals was approximately 65.5% of total mECM mass (Fig. S2B). The residual calcium and phosphate ions in mECM were quantified as 11.1 and 10.9 μg, respectively (Fig. S2C). During the mineralization process, the pH of the mixed PILP solution was monitored throughout the incubation period. The initial solution exhibited a pH of 7.8, which gradually decreased over time to pH 7.6 to 7.4 on day 1, pH 7.2 on day 4, and pH 7.0 by day 7. Meanwhile, the angiogenic factor profiles of ECM and mECM were found comparable to each other (Fig. 2J). Coagulation factor III [tissue factor (TF)], dipeptidyl peptidase IV (DPPIV), insulin-like growth factor-binding protein-1 (IGFBP-1), platelet-derived growth factor-AA (PDGF-AA), and Serpin E1 were detected in both ECM and mECM. In contrast, tissue inhibitor of metalloproteinase 1 (TIMP-1) was only confirmed in ECM. Densitometric quantification further confirmed mild reductions in the DPPIV and IGFBP-1 level, whereas TIMP-1 and PDGF-AA showed noticeably reduced level in mECM compared with ECM (Fig. S2D).

**Table 2.** Percentage of atomic compositions determined by XPS survey spectra

Samples	Atomic %					
	C 1s (%)	N 1s (%)	O 1s (%)	Ca 2p (%)	P 2p (%)	Ca/P
ECM	67.26	15.02	17.36	0	0.36	0
mECM	58.49	11.39	24.47	3.24	2.41	1.3444

**Table 3.** Elemental weight percentages determined by SEM–EDS analysis

Samples	Weight %		
	Ca K (%)	P K (%)	Ca/P
ECM	0	0.6	0
mECM	2.8	2	1.4

### Biocompatibility and cellular activation of mECM

#### Optimal dose dependence of mECM

The optimal concentration of mECM was determined by evaluating the cytotoxicity and osteogenic differentiation of mouse preosteoblasts (Fig. S3). Following mECM treatment, cells remained well attached and appeared more confluent in the low-dose mECM groups (1, 5, and 10 μg/ml) compared with untreated control group (0 μg/ml) (Fig. S3A). However, in the high concentrations of mECM (50 and 100 μg/ml), reduced cell confluence was partially observed, and cells appeared detached or aggregated together with mECM particles. Consistently, cell proliferation increased with mECM concentrations up to 10 μg/ml; however, the 50 and 100 μg/ml mECM groups showed lower OD values than the 10 μg/ml mECM group (Fig. S3B). In particular, 100 μg/ml mECM showed similar OD value to the untreated group. Osteogenic differentiation showed noticeable differences at and above mECM 10 μg/ml. At day 11, the untreated (0 μg/ml) and low-dose mECM group (1 and 5 μg/ml) exhibited no positive ARS staining, whereas the 10 μg/ml mECM group showed distinct red-stained regions (Fig. S3C). The high-dose mECM group (50 and 100 μg/ml) showed stronger positive staining. However, detached or aggregated cells were observed, and gross images revealed partially empty regions. Consistently, the OD values from ARS quantification also significantly increased at and above 10 μg/ml mECM, with markedly higher values measured in the high-dose groups (Fig. S3D). Thus, an optimal concentration of 10 μg/ml of mECM was determined based on dose-dependent analysis and used for further in vitro studies.

#### Effects of mECM on cell viability and proliferation

**Fig. 3.**
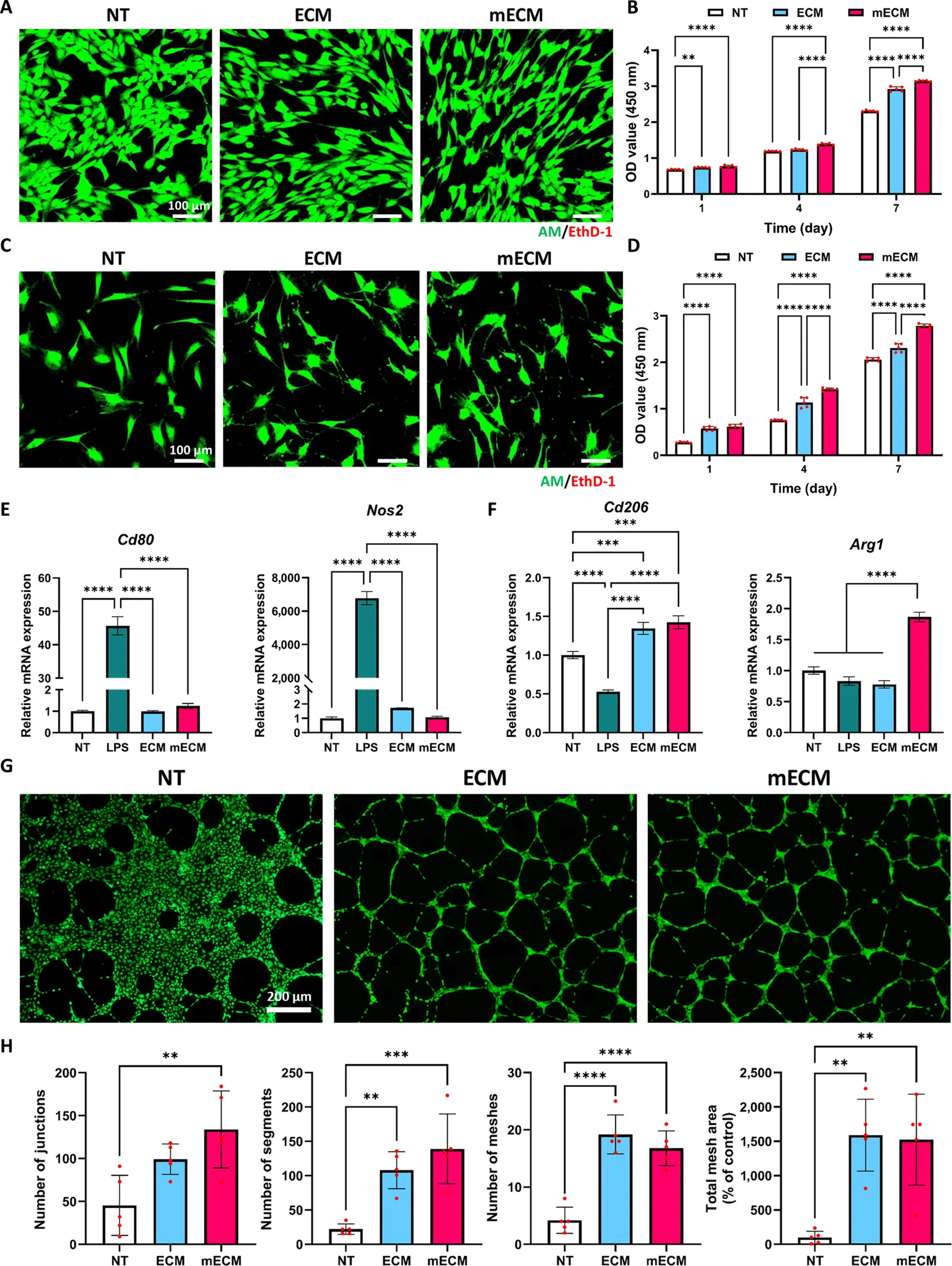
Biocompatibility and cellular activation of mECM (A) Fluorescence images of live (green) and dead (red) MSCs cultured with ECM and mECM, compared to the NT on day 3. Scale bar, 100 μm. (B) Proliferation of MSCs cultured with ECM and mECM for up to 7 d (*n* = 5, per group). (C) Live/dead assay images of MC3T3-E1 cells on day 3 following treatment with ECM and mECM. Scale bar, 100 μm. (D) CCK-8 assay demonstrating proliferation of MC3T3-E1 cells treated with ECM or mECM for up to day 7 (*n* = 5, per group). (E and F) Gene expression levels of (E) pro-inflammatory markers (*Cd80* and *Nos2*) and (F) anti-inflammatory markers (*Cd206* and *Arg1*) in RAW 264.7 macrophages treated with LPS, ECM, or mECM, normalized to NT (*n* = 3, per group). (G) Tube formation assay of HUVECs cultured with conditioned medium derived from the macrophages treated with ECM or mECM. Scale bar, 200 μm. (H) Quantitative analysis of tube formation, including the numbers of junctions, number of segments, number of meshes, and total mesh area (%) (*n* = 5, per group) (**P < 0.01, ***P < 0.001, ****P < 0.0001).

As the biocompatibility of ECM and mECM was evaluated using L&D staining and proliferation assays, hMSCs on ECM and mECM showed no detectable dead cells, and their morphology of cells was comparable to that of the NT group (Fig. 3A). The cell proliferation was faster in the mECM-treated group than in the NT group (Fig. 3B). Similarly, mouse preosteoblasts (MC3T3-E1) exhibited no dead cells in all test groups (Fig. 3C) and showed the highest level of cell proliferation in the mECM group (Fig. 3D).

#### mECM induced macrophage polarization toward anti-inflammatory phenotype

Macrophage polarization was assessed by evaluating the gene expression of pro- or anti-inflammatory markers. While LPS treatment could significantly elevate M1 markers (*Cd80* and *Nos2*), there was no significant increase of M1 marker expression in the ECM- and mECM-treated group (Fig. 3E). Instead, anti-inflammatory M2 markers (*Cd206* and *Arg1*) were significantly up-regulated in both ECM and mECM groups, especially higher expression of *Arg1* observed in the mECM (Fig. 3F). To evaluate their modulatory effects on LPS-induced inflammatory responses, cells were cotreated with LPS and either ECM or mECM (Fig. S4). The expression levels of M1 markers (*Cd80* and *Nos2*) were significantly lower in the LPS/ECM and LPS/mECM group compared to the LPS group, although they still remained higher than in the NT group (Fig. S4A). Conversely, the expression of M2 makers (*Cd206* and *Arg1*) in cotreated groups was significantly higher than in LPS (Fig. S4B).

#### Angiogenic potential of ECM and mECM

We sought to evaluate whether CM derived from macrophages grown on the ECM or mECM could promote the formation of capillary-like structure of HUVECs in a tube formation assay. As shown in Fig. 3G, endothelial cells (ECs) cultured in the control medium (NT) failed to form coherent tubular networks, remaining as scattered individual cells or small aggregates. In stark contrast, ECs cultured in CM obtained from macrophages on both ECM and mECM groups self-organize well-developed and interconnected capillary-like networks. Quantitative analysis confirmed these observations (Fig. 3H). Key angiogenic parameters, including the number of junctions, number of segments, number of meshes, and the total mesh area, were all significantly increased in the ECM and mECM groups compared to the NT control (*P* < 0.01). Notably, the mECM group exhibited the highest levels for the number of junctions and segments.

### mECM accelerated osteogenesis of mouse preosteoblast

**Fig. 4.**
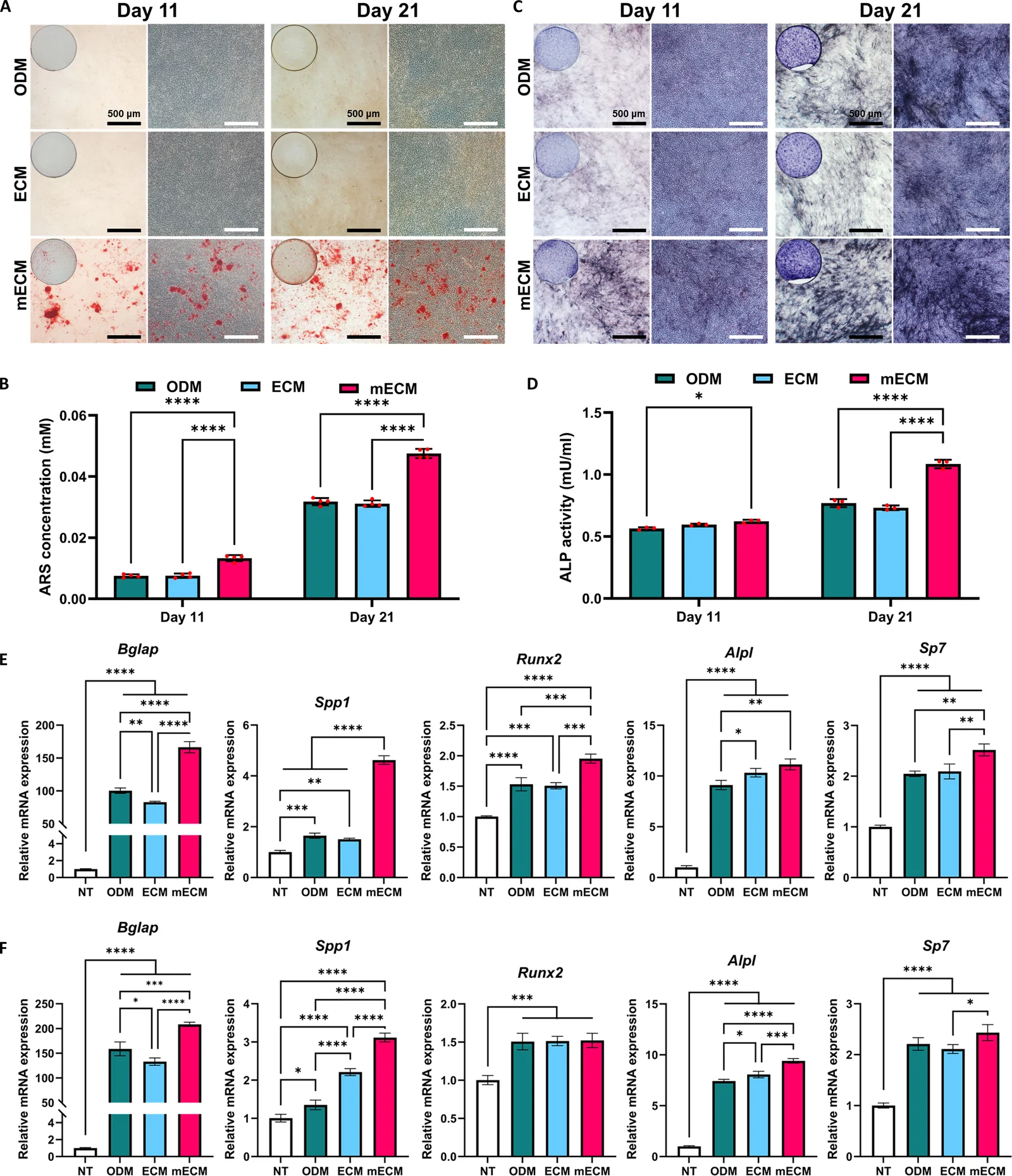
Effects of mECM on osteogenic differentiation of preosteoblasts. (A) ARS staining images of MC3T3-E1 cells at days 11 and 21 (left: bright field, right: phase contrast, inset: gross image of stained well). Scale bar, 500 μm. (B) Quantification of ARS concentration (mM) in differentiated MC3T3-E1 cells at days 11 and 21 (*n* = 4, per group). (C) ALP staining images of MC3T3-E1 at days 11 and 21 (left: bright field, right: phase contrast, inset: gross images of stained wells). Scale bar, 500 μm. (D) Quantitative analysis of ALP activity (mU/ml) in MC3T3-E1 cells at days 11 and 21 (*n* = 3, per group). (E and F) Relative mRNA expression levels of osteogenic markers: *Bglap* (OCN), *Spp1* (OPN), *Runx2*, *Alpl*, and *Sp7* (OSX) in MC3T3-E1 cells at (E) day 11 and (F) day 21 after induction of osteogenic differentiation in vitro (*n* = 3, per group) (*P < 0.05, **P < 0.01, ***P < 0.001, ****P < 0.0001).

Osteogenic differentiation of MC3T3-E1 was examined through chemical staining and gene expressions. ARS staining revealed calcium deposition in osteogenically induced cells. On day 11, positive ARS stains in red were observed only in mECM-treated cells. On day 21, weak red staining was observed in the ODM- and ECM-treated groups, whereas the mECM-treated group showed more pronounced increase in the number of stained cells (Fig. 4A). ARS quantification also showed that calcium deposition in the mECM-treated group was significantly higher than in the other groups all the time (Fig. 4B). The ALP activity is an indicator of osteogenic differentiation, as ALP-active cells are on their way to becoming osteoblasts. In ALP staining, there was no significant difference at early time point but the mECM-treated group exhibited a markedly higher number of ALP-positive cells on day 21 (Fig. 4C). Similarly, hMSCs cultured with mECM showed accelerated calcification and enhanced ALP activity, as confirmed by ARS and ALP staining, respectively (Fig. S5A and B). Quantitative analysis disclosed a similar trend; the mECM-treated group proved the highest activity at 21 d (Fig. 4D). In addition, significantly higher expression of osteogenic differentiation-related genes [*Bglap*, encoding OCN; *Spp1*, encoding osteopontin (OPN); *Runx2*; *Alpl*; and *Sp7*, encoding osterix (OSX)] was also confirmed with the mECM group at day 11 (Fig. 4E) and such expression pattern was maintained on day 21, except *Runx2* and *Sp7* (Fig. 4F).

### Fabrication of mECM sheet and the properties

**Fig. 5.**
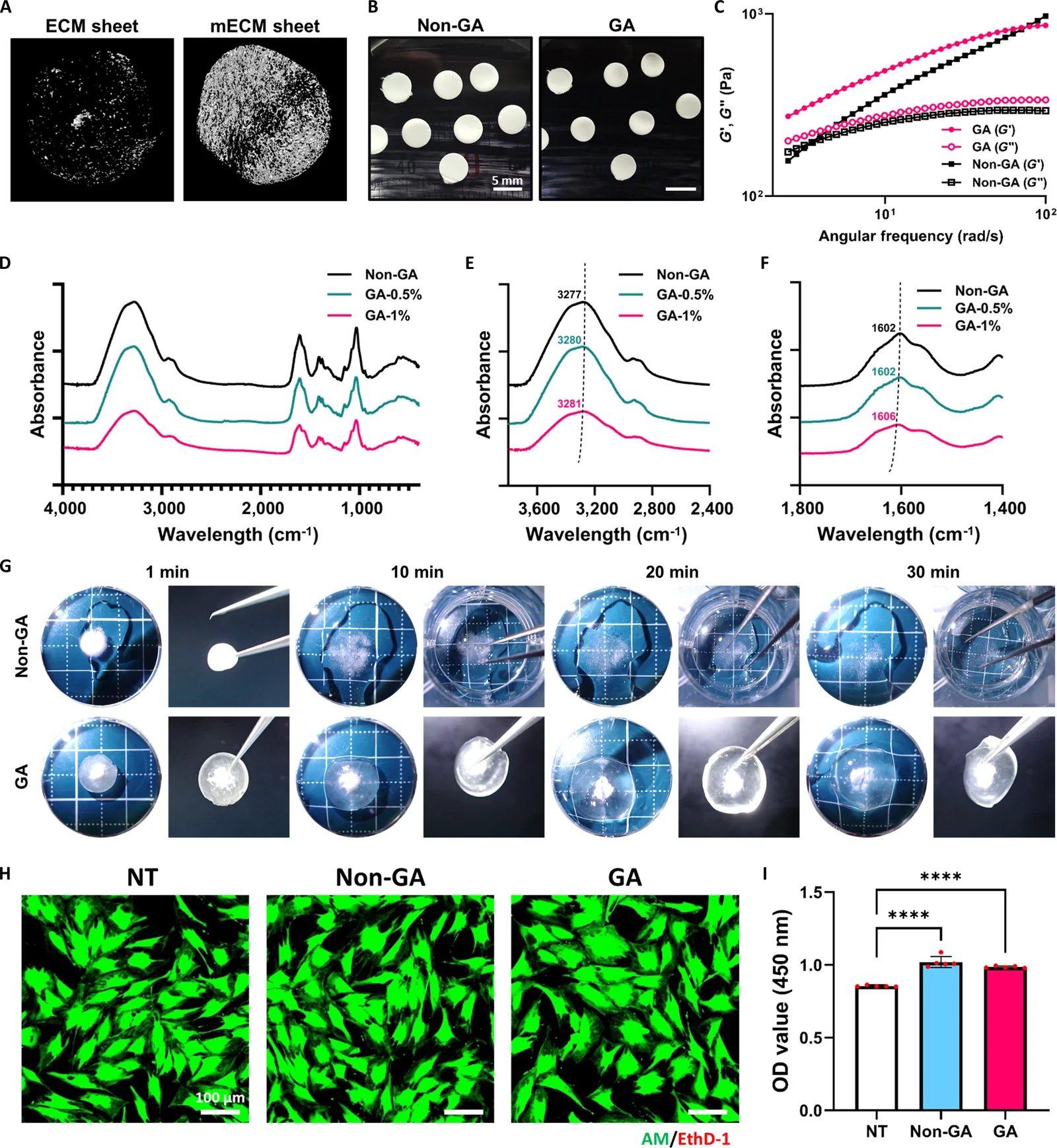
Fabrication of mECM sheet via GA crosslinking and its characteristics. (A) Representative micro-CT images of ECM and mECM sheet. (B) Representative images of mECM sheet before (Non-GA) and after GA crosslinking. Scale bar, 3 mm. (C) Rheological analysis of hydrated mECM sheets showing elastic (*G*′) and viscous (*G*″) moduli of GA (pink) and Non-GA (black) group. (D) FT-IR spectra of Non-GA (black), GA-0.5% (green; ECM 0.5%), and GA-1% (pink; ECM 1%) sheets. (E) Enlarged FT-IR spectra in the range of 4,000 to 2,400 cm^−1^ showing characteristic peaks at 3,277 (Non-GA), 3,280 (GA-0.5%), and 3,281 (GA-1%) cm^−1^. (F) FT-IR spectra in the range of 1,800 to 1,400 cm^−1^ exhibiting characteristic peaks at 1,602 cm^−1^ (Non-GA), 1,602 cm^−1^ (GA-0.5%), and 1,606 cm^−1^ (GA-1%). (G) Representative images of swelling behavior of Non-GA and GA mECM sheets at 1, 10, 20, and 30 min display their appearances and handling with forceps. (H) Live (green)/dead (red) fluorescence images of MC3T3-E1 cells cultured with Non-GA and GA sheets compared to NT control at day 3. Scale bar, 100 μm. (I) CCK-8 assay results of MC3T3-E1 cells treated with each sheet at day 3 (*n* = 5, per group) (****P < 0.0001). In GA-X%, X indicates the concentration of ECM used in the fabrication of mECM, not that of GA solution.

For in vivo transplantation, the ECM and mECM sheet were fabricated by mixing with HA, followed by crosslinking of amine and aldehyde groups through GA vapor treatment (Fig. 1). The mECM/HA solution was homogenously mixed, casted into molds, and subsequently freeze-dried, enabling the fabrication of mECM sheets with dimensions of 8 × 6 cm^2^ (Fig. S6A). As shown in viscosity measurements, viscosity increased with HA concentrations, and the solutions mixed with mECM exhibited slightly lower viscosity at the same HA concentration (Fig. S6B). In addition, quantitative assessment of the residual GA via MBTH assay showed only trace level of residual GA, with the concentrations of 3.61, 3.69, and 3.08 ppm for HA, ECM, and mECM sheets, respectively (Fig. S6C). For assessment of mineral deposition in the HA-added ECM or mECM scaffolds, micro-CT analysis showed that the ECM sheet was almost entirely radiolucent, indicating an absence of mineral content (Fig. 5A). In sharp contrast, the mECM sheet exhibited a uniformly distributed, high-density radiopaque signals throughout its structure, which clearly demonstrated the successful and homogeneous deposition of minerals in the scaffold. After GA-induced crosslinking (GA), the size of the ECM sheet slightly decreased compared to the non-crosslinked sheet (Non-GA) (Fig. 5B). Rheological analysis revealed that the GA maintained a stable solid-like behavior (*G*′ > *G*″) up to 100 rad/s, whereas the Non-GA exhibited liquid-like behavior below 10 rad/s but gradually increased up to 100 rad/s (Fig. 5C). FT-IR spectra of Non-GA, GA-0.5% (0.5% ECM), and GA-1% (1% ECM) samples discovered molecular compositional changes depending on the degree of crosslink (Fig. 5D). The peak at 3,277 cm^−1^ in the Non-GA sample, corresponding to the primary amine (N–H stretches), was shifted and broadened in GA-0.5% (3,280 cm^−1^) and GA-1% (3,281 cm^−1^) (Fig. 5E). Similarly, the peak at 1,602 cm^−1^ in the Non-GA sample, corresponding to the primary amine (N–H bend), was shifted and broadened in GA-1% (1,606 cm^−1^), while GA-0.5% (1,602 cm^−1^) showed only broadening (Fig. 5F). In the swelling test, the Non-GA sheet began to dissolve within 10 min but the GA sheet remained stable for up to 30 min and could even be held with forceps (Fig. 5G). After GA crosslinking, most cells cultured with sheets remained viable after 3 d of culture (Fig. 5H). The proliferation rate was also comparable to that of the nontreated (NT) group on day 3 (Fig. 5I).

### mECM sheet enhanced bone regeneration in vivo

**Fig. 6.**
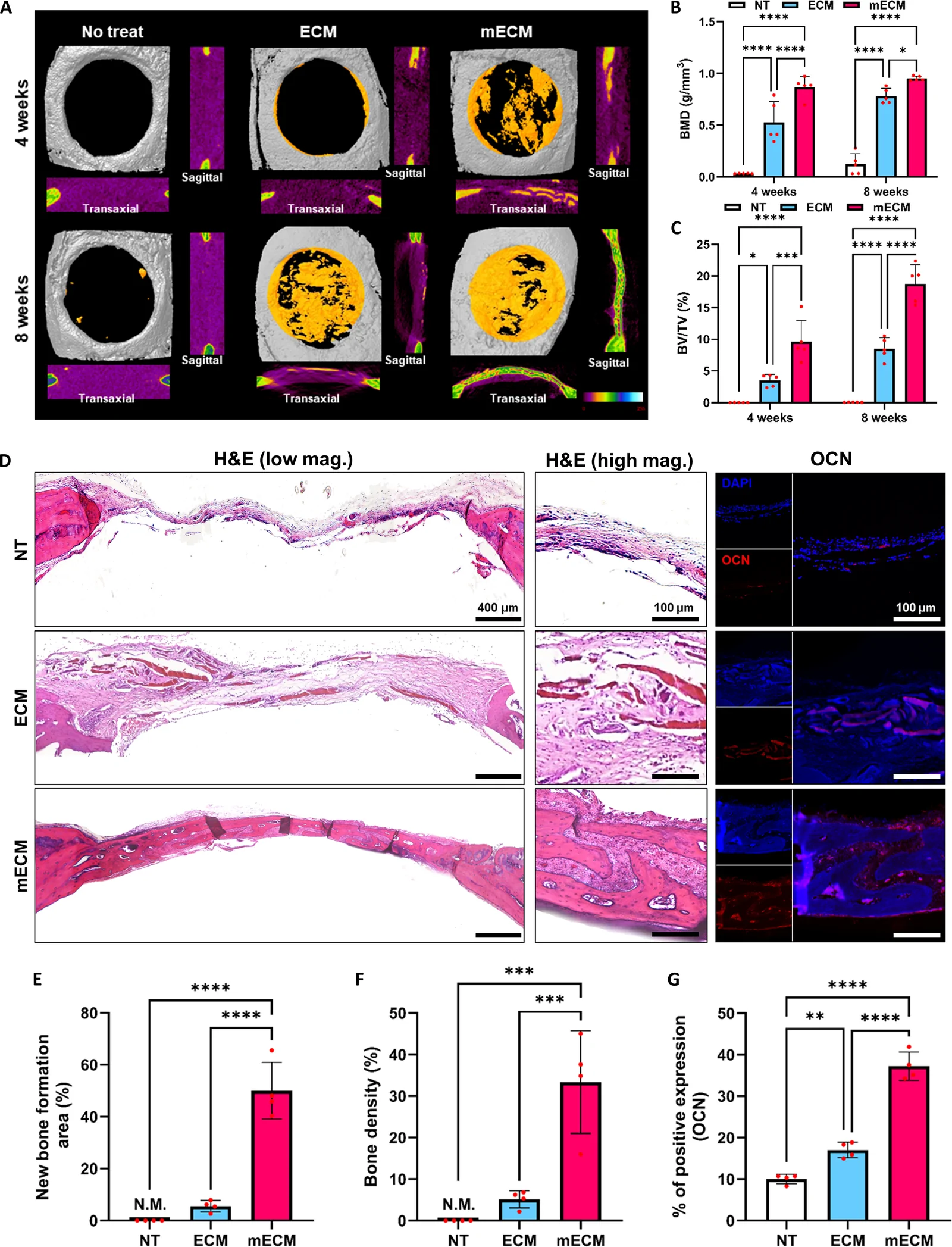
Bone regeneration of mECM in a mouse calvarial defect model. (A) Representative micro-CT images 3D reconstructed in transaxial and sagittal views of the calvarial defects at 4 and 8 weeks post-surgery. Newly formed bone is highlighted in yellow. The same animals were used for longitudinal micro-CT analysis at 4 and 8 weeks, respectively. For subsequent histological and immunohistochemical analyses, samples were randomly assigned and evaluated in a blinded manner. (B and C) Quantitative analysis of (B) BMD and (C) BV/TV from micro-CT scans (*n* = 5, per group). (D) Histological analysis of the defect sites at 8 weeks: H&E staining in high and low magnification (scale bar, 400 and 100 μm) and OCN immunofluorescence staining (red; scale bar, 100 μm). (E to G) Quantitative results of (E) new bone formation area (%), (F) bone density (%), and (G) OCN-positive expression (%) (*n* = 4, per group). Data are presented as mean ± SD (*P < 0.05, **P < 0.01, ***P < 0.001, ****P < 0.0001).

Upon the promising in vitro results that demonstrated the high osteogenic potential of our scaffolds, we next evaluated the in vivo bone regeneration efficacy in a mouse calvarial defect model. The progression of new bone formation was longitudinally monitored using micro-CT at 4 and 8 weeks. The reconstructed micro-CT images in Fig. 6A revealed that the defect in the nontreated, control group (NT) remained largely empty at both time points. In the ECM group, limited bone ingrowth originating from the defect margins was observed, and this ingrowth was insufficient to bridge the gap even at 8 weeks. Surprisingly, the mECM group disclosed substantial new bone formation as early as 4 weeks. The newly formed bone had almost completely bridged the defect, indicating superior regenerative capacity at 8 weeks. These findings were strongly supported by quantitative analysis. As shown in Fig. 6B and C, both the BMD at 4 and 8 weeks and the new bone volume (BV/TV) at 4 and 8 weeks were remarkably highest in the mECM group compared to the other groups. The difference was statistically significant (*P* < 0.0001). The sustained increase of bone volume in the mECM group suggested continuous osteogenic activity over time. When the harvested samples at 8-week post-transplantation were subjected to histological analysis, the H&E staining showed that the defect in the NT group was filled primarily with non-ossified fibrous connective tissue (Fig. 6D). In the ECM group, several isolated bone spicules were observed within the defect but these were insufficient to form a continuous bone bridge. Conversely, the mECM group exhibited complete osseous bridging with mature bone tissue and clearly showed well-integrated osteocytes within the bone matrix in high-magnification images. In addition, Herovici’s staining revealed a higher abundance of mature collagen fibers in the mECM-treated group (Fig. S7). To identify mature osteoblasts, immunofluorescence staining for OCN, a late-stage marker of bone formation, was carried out. While OCN expression was minimal in the NT group, the ECM group displayed a notable increase in OCN-positive signals (Fig. 6D). This expression was further amplified throughout the newly formed bone in the mECM group. Moreover, quantitative analysis was also supportive of these observations, where the new bone area, bone density, and OCN-positive area were all significantly greater in the mECM group than in the NT and ECM control (*P* < 0.0001) (Fig. 6E to G). These results collectively demonstrate that the mECM scaffold not only induced a large quantity of new bone formation but also promoted its maturation into a high-quality bone tissue.

### ECM and mECM sheets promote early angiogenesis and pro-regenerative immune response

**Fig. 7.**
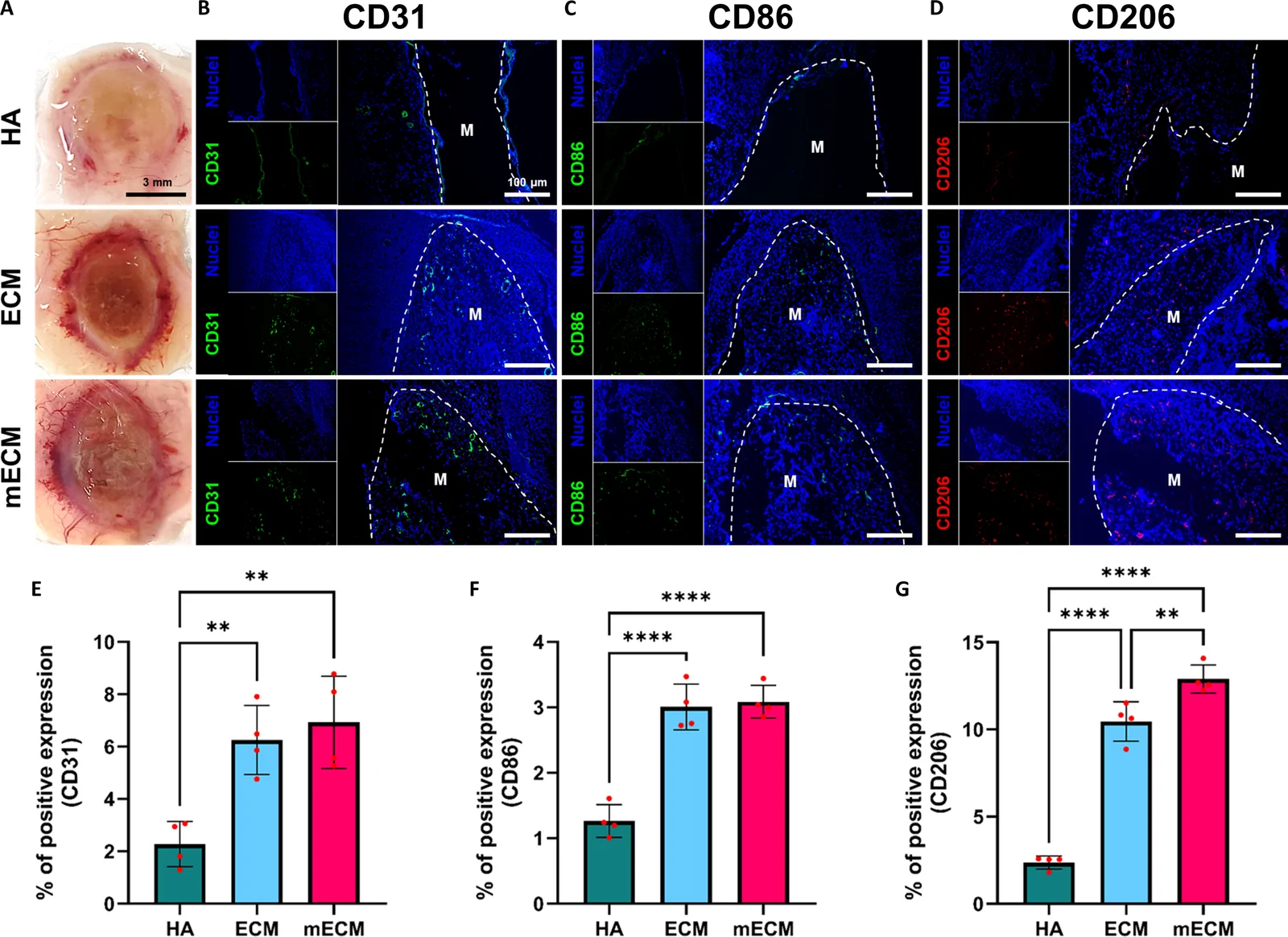
Examination of angiogenesis and macrophage polarization of defect sites at early time point. (A) Gross morphology of harvested materials (HA, ECM, and mECM). Scale bar, 3 mm. (B to D) Immunofluorescence staining of the surrounding tissue for (B) angiogenesis marker CD31 (green), (C) M1 macrophage marker CD86 (green), and (D) M2 macrophage marker CD206 (red). Nuclei were counterstained with DAPI (blue). The dotted line marks the interface between the material (M) and the host tissue. Scale bar, 100 μm. (E to G) Quantitative analysis of the positive area (%) for each marker [(E): CD31, (F): CD86, (G): CD206] obtained from the representative sections (*n* = 4, per group). Data are presented as mean ± SD (**P < 0.01, ****P < 0.0001).

Gross observation of transplantation at 1 week (Fig. 7A) showed that the implantation sites in both the ECM or mECM exhibited a distinct reddish coloration, suggesting ongoing neovascularization inside the sheets, which was further confirmed by staining for the endothelial cell marker CD31 (green) (Fig. 7B). In contrast, the HA treated group displayed a pale appearance in gross image and showed few CD31-positive signals in histological sections. Next, we investigated the early immune response, particularly the role of macrophages (Fig. 7C and D). Pro-inflammatory M1 macrophages (CD86) were found in all groups but were more prominent in the ECM and mECM group, indicating an initial recruitment of immune cells. More importantly, the expression of pro-regenerative and anti-inflammatory M2 macrophages (CD206) was markedly different. While barely detectable in the HA group, a high level of M2 macrophage infiltration was observed in both ECM and mECM group. Quantitative analysis further confirmed these results, showing that the positive area of CD31 (endothelial cells), CD86 (M1 macrophages), and CD206 (M2 macrophages) were all significantly higher in the ECM and mECM groups compared to the HA control (*P* < 0.01) (Fig. 7E to G).

## Discussion

Regarding the cell sources in this study, while bone marrow MSCs are traditionally favored for bone tissue engineering, due to their osteogenic potential, they typically exhibit a strict contact inhibition during culture, which inherently limits large-scale ECM production. Conversely, UCMSCs maintain active proliferation and form dense cell aggregates at high confluence, resulting in significantly enhanced endogenous ECM production [15]. Our primary interest was to obtain an abundant ECM matrix to serve as a sufficient substrate for mineralization. Moreover, our main objective was to investigate whether minerals formed on the ECM could instruct host osteogenesis, rather than relying on the innate osteogenic potential of the ECM itself. Therefore, UCMSC-derived ECM was considered an appropriate candidate because it enabled efficient ECM production while retaining bioactive properties suitable for a comparative analysis between unmineralized ECM and mECM.

The PILP mineralization strategy has been widely applied to collagen sponges, acrylamide-based scaffolds, polycarbonate membranes, and decellularized tissues to recapitulate the nanostructural and compositional characteristics of native bone [6]. In the PILP process, negatively charged polymers stabilize ACP as a liquid-like precursor, preventing premature crystallization. This fluidic precursor infiltrates the intrafibrillar spaces of fibers via capillary forces, where it subsequently transforms into oriented CaP nanocrystals by stabilizing ACP precursors [19,20]. We utilized the PILP mineralization strategy on cdECM to stabilize ACP precursors and promote matrix-associated mineralization in this study. Optimization of PILP mineralization was evaluated based on mineral content and ECM stability to maximize the minerals trapped in the cdECM, and the optimal condition was determined to be 10× concentration during 1 week of incubation (Fig. S1). In microscopic images at 3 weeks, the observation of aggregated minerals suggested the transition of amorphous minerals into crystalline HAp. On the other hand, the cdECM was unstable under other conditions; particularly, the 1× concentration exhibited a substantial loss of ECM proteins (Fig. S1D and E). Although the acquisition of fully mature crystalline HAp was beyond the scope of this study, our findings indicate that PILP can be applied to cdECM for homogeneous and metastable CaP mineralization while preserving the biochemical integrity of the ECM under the optimized condition.

Under the optimized condition, stable and homogeneous CaP mineral deposition was achieved, as extensively confirmed by various analytical instruments (Fig. 2A to I). A minor phosphate signal detected in the untreated ECM was likely attributable to residual components originating from the culture medium or PBS. The Ca/P ratio of minerals deposited on the ECM was confirmed by XPS atomic analysis and EDS elemental weight percentages, revealing that the mineral phase consisted of ACP (Ca/P ratio: 1.2 to 1.5). ACP represents a noncrystalline, highly hydrated transient precursor of CaP [9]. While crystalline HAp is commonly utilized as a bone substitute, ACP lacks a rigid crystal lattice and therefore exhibits higher thermodynamic solubility and faster resorption [21]. This property enables synchronized degradation with host cell infiltration, promoting the localized release of calcium and phosphate ions that serve as biochemical cues to stimulate osteogenic gene expression and enhance bone regeneration [22]. Consequently, ACP, the primary mineral constituent of mECM, represents a promising mineral phase for promoting bioactive bone regeneration, offering distinct advantages over conventional crystalline HAp. The CaP minerals generated via the PILP process were broadly distributed throughout the ECM, particularly along fibrillar structures (Fig. 2H and I), a pattern consistent with previous reports of PILP-mediated mineralization in collagen matrices [20,23]. When the mECM was examined across different incubation periods by SEM images, the ECM fibers gradually became thicker and covered with more minerals (Fig. S2A). Such physical accumulation adds substantial volumetric thickness and topographical depth to the underlying matrix framework. Consequently, this continuous buildup transformed the thin, flat matrix layout into the visually pronounced 3D microstructural network observed under SEM. Quantitative analysis demonstrated that the mineral phase accounted for approximately 65% of the mECM mass (Fig. S2B). Residual ions were detected at approximately 2.6% of the total mineral content in the mECM (Fig. S2C). These findings suggest that residual ions may exert a limited influence during the initial stages of biological assay and thus are unlikely to substantially affect long-term outcomes. We also carefully assessed whether the mineralization process compromised the diverse bioactive molecules in the cdECM, including angiogenic factors. A slight reduction was observed in a few factors such as IGFBP-1 and DPPIV, whereas TIMP-1 and PDGF-AA were more notably reduced in the mECM compared to the native ECM (Fig. 2J and Fig. S2D). Although the mineralization protocol was carefully executed to preserve protein integrity, this moderate loss of soluble factors occurred, because the process requires an extended incubation period and multiple washing steps. Collectively, our approach establishes the PILP-based mineralization as a promising strategy for engineering cdECM-based scaffolds with enhanced CaP mineral content for the applications in hard tissue regeneration.

For in vitro study, the optimal concentration of mECM was determined through dose-dependent analyses of cytotoxicity and osteogenesis (Fig. S2). All tested mECM concentrations exhibited no apparent cytotoxicity, and cell proliferation appeared to concentration-dependent manner up to 10 μg/ml mECM. However, high concentrations (50 and 100 μg/ml) of mECM showed abnormal cell growth behaviors, including partial cell detachment and aggregation with mECM particles (Fig. S2A and B). These results suggest that high-dose mECM treatment may not provide reliable experimental outcomes for long-term or stable 2D culture. Although osteogenic activity appeared to increase at and above 10 μg/ml mECM, cells treated with high concentrations of mECM tended to form aggregate and became trapped within mineral deposits (Fig. S2C and D). In addition, RNA extraction from high-dose mECM groups was technically challenging, likely due to the excessive CaP content. Previous studies have demonstrated that CaP minerals can interfere with RNA isolation through chelation effects, including the physical trapping of cellular material and the coprecipitation of ions with RNA [24,25]. Taken together, considering both the biological effects and technical limitations, 10 μg/ml mECM was determined as the optimal concentration and used for subsequent in vitro studies.

Biological properties of biomaterials are also an important aspect in coordinating diverse cellular responses, especially when transplanted into the in vivo milieu. As such, we comprehensively evaluated the mECM in terms of cytocompatibility, macrophage response, and angiogenic potential (Fig. 3). Both the native ECM and mECM showed no signs of cytotoxicity, and the mECM-treated group disclosed significantly enhanced cellular proliferation (Fig. 3A to D). Although the biocompatibility of ECM is well established, our results confirmed that the mECM likewise provides a favorable microenvironment for cellular behaviors. Notably, data obtained from mouse preosteoblasts suggest that the CaP-mineralized ECM provides biochemical and structural cues that promote osteogenic cell proliferation. In addition, polarization of host macrophages toward an anti-inflammatory M2 phenotype is critical for mitigating chronic inflammation and accelerating tissue repair [26]. Macrophage interactions with native collagen fibrils and associated glycosaminoglycans abundantly present within the ECM would suppress classical M1 activation and favor a pro-resolving phenotype [27,28]. In agreement with these reports, our previous studies demonstrated that cdECM promoted macrophage polarization toward an anti-inflammatory M2-like phenotype [29,30]. The mECM scaffold preserves these intrinsic matrix–integrin interactions, along with additional mineral-derived ions. Early studies showed that released extracellular Ca^2+^ can modulate macrophage behavior through calcium-sensing receptor (CaSR)-mediated signaling, potentially activating downstream Wnt/β-catenin-associated pathways that promote polarization toward an anti-inflammatory M2-like phenotype [31,32]*.* The mECM treatment was also associated with increased expression of M2-associated markers, including Cd206 and Arg1 (Fig. 3F). Furthermore, macrophages cultured with either ECM or mECM exhibited lower M1 and higher M2 markers even under inflammatory conditions induced by LPS (Fig. S3). M2 macrophages would regulate ECM remodeling and coordinate the balance between osteoblast and osteoclast activity, thereby facilitating constructive bone regeneration [33,34]. Adequate angiogenesis is also essential for successful bone regeneration to ensure sufficient blood supply to the defect site. Given that angiogenesis is strongly influenced by macrophage-derived cytokines [35,36], investigation of in vitro angiogenic potential of ECM and mECM using macrophage-mediated assay disclosed that the CM collected from macrophages treated with either ECM or mECM could significantly accelerate HUVEC tube formation (Fig. 3G to I). The transition toward an M2-like macrophage phenotype is a critical step in initiating tissue repair and orchestrating regeneration, as these macrophages establish a pro-regenerative microenvironment through the secretion of potent angiogenic factors, including vascular endothelial growth factor (VEGF), basic fibroblast growth factor (bFGF), and PDGF [37]. Accordingly, the observed enhancement in HUVEC tube formation is consistent with those of the angiogenic secretomes of M2-polarized macrophages [26]. This phenomenon aligns with our previous findings that cdECM was associated with the induction of pro-regenerative cytokines release from macrophages [29,30].

In fact, the osteoinductive property is considered one of the most crucial qualities for bone regenerative material. Accordingly, the osteoinductive capacity of the mECM was further evaluated using an in vitro osteogenic differentiation model (Fig. 4). Biomimetic materials designed for bone repair are expected to be biocompatible and to provide microenvironmental cues that promote osteogenic differentiation. The mECM satisfied these criteria, as evidenced by significantly enhanced calcification and ALP activity (Fig. 4A to D). Typically, MC3T3-E1 cells initiate calcification after approximately 20 d of osteogenic induction. However, MC3T3-E1 cells cultured with mECM showed markedly early initiation of calcification. This calcium deposition was originated from the cells rather than the material itself, as mECM without cells exhibited no detectable mineral accumulation (data not shown). Enhanced ALP activity further indicates that mECM accelerates early osteogenic commitment by providing bone-like biochemical and structural cues. These results were corroborated in hMSCs (Fig. S5), which similarly revealed earlier calcification and enhanced ALP activity in response to mECM compared with other groups. These findings suggest that the CaP minerals incorporated within mECM may partially dissolve and release bioactive ions that function as osteogenic cues, as documented in previous studies [22]. In fact, released Ca^2+^ or PO_4_^3−^ ions have been reported to stimulate distinct intracellular signaling pathways [38,39]. These pathways may cooperatively promote the transcription of osteogenic markers such as ALP and OPN, thereby facilitating bone matrix mineralization. Mature osteoblast markers, OCN and OPN, respectively, also recorded the highest gene expression level in the mECM-treated group at both time points in vitro (Fig. 4E and F). Moreover, a key transcription factor, OSX, that regulates osteogenic markers, such as ALP, OCN, and OPN, was also up-regulated in the mECM-treated cells, further supporting the enhanced osteogenic potential of mECM. Meanwhile, the notable in vivo performance of the mECM sheet may be explained by a synergistic interplay. Although the key mechanism behind mECM function in vivo is not thoroughly investigated in this work, it is plausible that biological efficacy of mECM might be multifaceted, enabling the coordinated activations of several key molecular pathways, for example, osteogenesis [40], immunomodulation [41], and angiogenesis [42,43]. Such interplay between mineral-derived osteogenic activity and cdECM-derived biochemical cues may underlie the regenerative potential of our mECM scaffold (Figs. 6 and 7).

For in vivo transplantation, we fabricated sheet-type mECM scaffolds and enhanced their physical stability via GA vapor crosslinking (Fig. 1 and Fig. S6). HA, a naturally derived and biocompatible polymer, was selected as the supporting material [44]. Because HA alone lacks inherent osteoinductivity, it was combined with mECM to provide osteoinductive functionality while compensating for the limited physical stability of mECM. HA was fully dissolved in both ECM and mECM solutions, facilitating molding by increasing solution viscosity and stabilizing the pre-sheet structure after freeze-drying (Fig. S6A). Although higher HA concentrations were technically achievable, homogeneous mixing with the mECM solution became difficult, resulting in phase separation at concentrations above 3%. Biomaterial scaffolds require sufficient physical stability to maintain structural integrity during the early healing phase and to support localized retention of bioactive cues at the defect site. In this study, GA vapor crosslinking was employed to enhance the structural stability of the mECM sheet through imine bond formation between aldehyde groups and naturally abundant amine groups present in the ECM proteins and glycosaminoglycans [45]. Our mECM sheet is intended as a temporary bioactive scaffold rather than a structural bone substitute. Although the present rheological analysis does not directly assess load-bearing properties such as compressive strength or tear resistance, it demonstrated sufficient physical stability under hydrated conditions, as evidenced by stable solid-like viscoelastic behavior (Fig. 5C). Notably, the non-crosslinked sheets rapidly dissolved under aqueous conditions, whereas the crosslinking via GA vapor exposure (15 h) provided not only sufficient physical stability but good cell viability (Fig. 5G). Given the well-documented cytotoxicity associated with residual GA, particular attention was paid to evaluating post-processing biocompatibility. Fortunately, all sheet groups, including the GA-crosslinked sheets, exhibited no measurable cytotoxicity (Fig. 5H and I), indicating effective removal of the residual GA and preservation of cytocompatibility. While there are other crosslinking methods, such as 1-ethyl-3-(3-dimethylaminopropyl)carbodiimide (EDC)/*N*-hydroxysuccinimide (NHS) or genipin, the primary reason we selected GA is that we have long, practical experiences of crosslinking biomaterials via GA vapor. Collectively, these results demonstrate that GA crosslinking enabled fabrication of a physically stable sheet-type mECM scaffold suitable for in vivo delivery of ECM-derived biological cues and mineral components to support new bone formation.

When we assessed the bone regeneration capability in a mouse calvarial defect model (Fig. 6), the mECM scaffold supported significantly greater bone regeneration than native ECM, underscoring the importance of combining an organic ECM framework with a mineral component. Because the cdECM was derived from human cells, its xenogeneic application in a rodent model raised potential immunogenicity concerns. However, the decellularization process is specifically designed to remove major immunogenic cellular components, which serve as the primary triggers of acute host immune rejection [13,46]. Additionally, numerous ECM components are structurally conserved across mammalian species, enabling cross-species biological interaction with relatively low immunogenicity following appropriate decellularization [47,48]. While HA primarily served as a structural support scaffold, its direct osteogenic contribution was minimal under the present experimental setting. This is further corroborated by our early-stage host response analysis (Fig. 7), in which the HA group showed limited recruitment of pro-regenerative M2 macrophages and failed to induce robust angiogenesis. Therefore, the enhanced bone formation of mECM group is more likely attributable to the microenvironment provided by mECM, enriched with CaP minerals and ECM-derived biochemical cues, rather than to the HA component itself. The early-stage in vivo results also provided evidence of the structural stability of mECM sheets, as the interface between the host tissue and the scaffold remained well-defined. Although a precise quantitative degradation assay was beyond the scope of the current study, the in vivo observations (Figs. 6 and 7) suggested that GA crosslinking effectively prevented premature degradation, thereby providing a regenerative niche that facilitated cellular infiltration and angiogenesis. In fact, synchronization between scaffold degradation profile and new bone formation rate is a fundamental aspect for functional bone regeneration, which was not fully evaluated in this study. It is notable, however, that it is of great importance for complete tissue regeneration but extremely challenging in precisely controlling such events in vivo. Meanwhile, the relationship between mineral content and osteogenic outcomes was not systematically evaluated in this study. Excessive mineralization may reduce matrix bioactivity, whereas insufficient mineralization can limit osteoinductive effects. Since this study primarily focused on establishing homogeneous ACP mineralization while preserving cdECM integrity, future studies should investigate the effects of varying mineral contents on scaffold degradation, ion release, and osteogenic differentiation to further optimize regenerative performance.

In conclusion, this study demonstrates that our mECM and mECM sheet provide bioactive microenvironments that effectively support not only osteogenic differentiation in vitro but also bone regeneration in vivo. As a technical advancement, the integration of cdECM with PILP-mediated mineralization successfully yielded a novel mECM scaffold capable of delivering ECM-derived bioactive factors together with CaP mineral components. The mECM scaffold retained excellent biocompatibility and bioactivity, promoting both osteogenesis and angiogenesis. Collectively, our findings suggest that mECM scaffold represents a promising next-generation biomaterial platform for bone tissue regeneration.

## Data Availability

The datasets used and/or analyzed during the current study are available from the corresponding authors on reasonable request.
