# Supplementary material for: Bioactive Mineralized Cell-Derived Extracellular Matrix via Polymer-Induced Liquid Precursor Enhances Osteogenesis and Bone Regeneration
Source: Biomater Res. 2026 Sep 1;30:0410. doi: 10.34133/bmr.0410 (PMC13530373; doi:10.34133/bmr.0410)
Supplement: Supplementary file 1 [file bmr.0410.f1.docx]

**Supplementary Data**

**Title:** Bioactive Mineralized Cell-Derived Extracellular Matrix via Polymer-Induced Liquid Precursor Enhances Osteogenesis and Bone Regeneration

**Authors:** Jae Won Kwon^1, #^, Jin Jeon^1, #^, Seung Won Yang^1, 2^, Youngdoo Chung^3^, Jin Hee Park^3^, Yoon Ki Joung^1, 2^, Hee Seok Yang^3,4, *^, and Kwideok Park^1, 2, *^

**Supplementary Figure 1.** Optimization of PILP mineralization on cdECM. (A) Representative macroscopic images of the mineralized ECM (mECM) across varying concentration factors (1X, 5X, 10X, and 20X) and incubation periods (1, 2, and 3 weeks). Scale bar=100 μm. Quantitative (B) calcium and (C) phosphate profiling of mECM within 6-well plates under each respective condition (n=3 per group). (D) Representative images highlighting localized regions of matrix detachment at 3 weeks. Scale bar=100 μm. (E) Relative total protein content (%) of mECM across all conditions, normalized to native ECM (n=3 per group).

**Supplementary Figure 2.** Detailed characterizations of mineralized ECM (mECM). (A) High magnifications (10000X and 50000X) of scanning SEM micrographs showing the surface morphology of mECM across different incubation periods (0, 1, 4, and 7 days). Scale bar=5 and 1 μm, respectively. (B) Pie chart illustrating the relative mass proportions of minerals, proteins, and unidentified constituents within the total mECM mass. (C) Quantitative assessment of residual ions (calcium and phosphate) leached from the mECM (n=3 per group). (D) Chemiluminescence density (%) of human angiogenic factors in the native ECM and mECM, normalized to the positive control (n=3 per group).

**Supplementary Figure 3.** Dose-dependence of mECM. (A) Representative light microscopy images of MC3T3-E1 cells treated with different concentrations of mECM (0, 1, 5, 10, 50, and 100 μg/mL). (B) Optical density (OD) values determined by CCK-8 assay of MC3T3-E1 cells under each condition (n=5 per group). (C) Alizarin Red S (ARS) staining images of MC3T3-E1 cells following 11days of treatment at each concentration, with inset showing gross images of the stained wells. Scale bar=200 μm. (B) Quantification of extracellular calcium deposition via ARS extraction, presented as OD values (at 405 nm) in differentiated MC3T3-E1 cells under each condition (n=5 per group).

**Supplementary Figure 4.** Macrophage responses under inflammatory conditions stimulated by LPS. Gene expression levels of (A) pro-inflammatory markers (Cd80 and Nos2) and (B) anti-inflammatory markers (Cd206 and Arg1) in RAW 264.7 macrophages treated with LPS, LPS/ECM, or LPS/mECM, normalized to untreated controls (NT) (n=3 per group).

**Supplementary Figure 5.** Osteogenic effects of mECM on human mesenchymal stem cell (hMSC). (A) Alizarin Red S (ARS) staining of hMSCs at days 3, 7 and 14 (inset: gross image of stained wells). Scale bar=200 μm. (B) Alkaline phosphatase (ALP) staining of hMSCs at day 3, 7, and 14 (inset: gross images of stained wells). Scale bar=200 μm.

**Supplementary Figure 6.** Fabrication process and characterizations of the mECM sheet. (A) Gross macroscopic images illustrating each step of the mECM presheet fabrication process (prior to crosslinking). Scale bar=2 cm. (B) Viscosity curves of pure hyaluronic acid (HA) solutions at different concentrations (1, 2, and 3 wt%) and the corresponding HA solutions blended with mECM suspensions. (C) Evaluation of residual glutaraldehyde (GA) concentration in the crosslinked hydrogel groups determined via MBTH assay (n=4 per group; data are presented in ppm as mean ± SD).

**Supplementary Figure 7.** Assessment of collagen maturation for each group in a calvarial defect model. Herovici’s staining images of the calvarial defects treated with ECM and mECM sheets at 8 weeks post-surgery. Scale bar=100 μm.
